# Flat electrode contacts for vagus nerve stimulation

**DOI:** 10.1371/journal.pone.0215191

**Published:** 2019-11-18

**Authors:** Jesse E. Bucksot, Andrew J. Wells, Kimiya C. Rahebi, Vishnoukumaar Sivaji, Mario Romero-Ortega, Michael P. Kilgard, Robert L. Rennaker, Seth A. Hays

**Affiliations:** 1 The University of Texas at Dallas, Erik Jonsson School of Engineering and Computer Science, Richardson, Texas, United States of America; 2 Texas Biomedical Device Center, Richardson, Texas, United States of America; 3 The University of Texas at Dallas, School of Behavioral Brain Sciences, Richardson, Texas, United States of America; Szegedi Tudomanyegyetem, HUNGARY

## Abstract

The majority of available systems for vagus nerve stimulation use helical stimulation electrodes, which cover the majority of the circumference of the nerve and produce largely uniform current density within the nerve. Flat stimulation electrodes that contact only one side of the nerve may provide advantages, including ease of fabrication. However, it is possible that the flat configuration will yield inefficient fiber recruitment due to a less uniform current distribution within the nerve. Here we tested the hypothesis that flat electrodes will require higher current amplitude to activate all large-diameter fibers throughout the whole cross-section of a nerve than circumferential designs. Computational modeling and in vivo experiments were performed to evaluate fiber recruitment in different nerves and different species using a variety of electrode designs. Initial results demonstrated similar fiber recruitment in the rat vagus and sciatic nerves with a standard circumferential cuff electrode and a cuff electrode modified to approximate a flat configuration. Follow up experiments comparing true flat electrodes to circumferential electrodes on the rabbit sciatic nerve confirmed that fiber recruitment was equivalent between the two designs. These findings demonstrate that flat electrodes represent a viable design for nerve stimulation that may provide advantages over the current circumferential designs for applications in which the goal is uniform activation of all fascicles within the nerve.

## Introduction

Vagus nerve stimulation (VNS) is one of the most widely used peripheral nerve stimulation strategies and has been employed in over 70,000 patients for control of epilepsy [1]. Recent clinical studies demonstrate the potential of VNS for treatment of other neurological disorders, including stroke, tinnitus, headache, and arthritis [2–5]. Given the broad potential applications, there is a great deal of interest in identifying optimal stimulation strategies to maximize benefits in patients [6].

Implanted helical cuff electrodes are the gold-standard method for VNS. Because of their role in seizure suppression, many current VNS applications seek to predominantly activate large diameter A-fibers [7]. Activation of smaller diameter fibers, such as B- and C-fibers, while desired for cardiovascular control in some applications, are typically avoided for VNS used for epilepsy. In order to maximize A-fiber activation, the recruitment curve should be as steep as possible, such that the majority of A-fibers are activated before the stimulation reaches intensities that will exceed the threshold for smaller diameter fibers. Steep recruitment curves have been observed with circumferential or helical electrodes that cover the majority of the circumference of the nerve, which suggests that although the current density decays from the edge of the electrodes to the center of the nerve, it is still relatively uniform.

Current VNS electrode designs are expensive to manufacture and can prove challenging to place on the nerve [8]. A flat configuration inside of an insulating cuff with electrode contacts on one side of the nerve could be fabricated with a more compact design that would simplify production and implantation. However, this electrode geometry provides contact with only a small portion of the circumference of the nerve, which will produce a less uniform current distribution than the standard helical design. The reduced surface area of a such a design will result in higher current density near the electrode and the larger distance between the electrode and the opposite side of the nerve will result in a greater decay and consequently lower current density. As a result, higher-threshold fibers near the electrodes may be activated before distant lower-threshold fibers. Many recent developments in peripheral nerve stimulation have taken advantage of this principle to achieve selective stimulation [9–11]. While emerging applications of VNS may ultimately benefit from selective stimulation, current applications of VNS primarily focus on uniform activation of A-fibers across the nerve [12]. Thus, a direct comparison of flat and circumferential cuff electrodes is needed to determine if flat contacts represent a practical alternative to provide steep recruitment of the fibers within the vagus nerve. In the present study, we performed modeling and empirical testing to examine the effect of varying the geometry of the electrode contacts on the efficiency of nerve recruitment in a number of conditions.

## Materials and methods

### Computational model

A 3D model was created in Comsol (COMSOL Multiphysics® Version 5.3) consisting of a nerve with a single fascicle, perineurium, epineurium, two platinum contacts, an insulating cuff, and ambient medium, similar to previous studies [13,14]. In a subset of models, a multi-fascicle nerve containing five fascicles was used. The nerve had a diameter of either 0.9 mm for the rat sciatic, 0.4 mm for the rat vagus, or 3 mm for the rabbit sciatic [15–17]. Perineurium thickness was set to 3% of the fascicle diameter [18]. Epineurium thickness was set to 0.13 mm for the rat sciatic, 0.1 mm for the rat vagus, and 0.43 mm for the rabbit sciatic [19–21]. To investigate the effect of nerve size, the rabbit sciatic model was scaled from 4 times smaller to 1.5 times larger. For both rat nerves, the insulating cuffs had an inner diameter of 1 mm and outer diameter of 2 mm. For the rabbit sciatic, insulating cuffs had an inner diameter of 3.02 mm and outer diameter of 5.2 mm. Platinum contacts had a thickness of 0.01 mm. Flat contacts used in the rabbit model had a width of 2 mm and length of 1.5 mm in the axial direction. The cross-sectional area of the nerve and of the cuff lumen was matched between the circumferential and flat electrode models by increasing the inner diameter of the flat cuff by 8.67%. The nerve was modified to take on the shape of the flat cuff [22]. Helical electrodes with a width of 0.7mm and thickness of 0.01mm had a pitch of 2 mm and completed a 270° arc. The insulation had the same pitch and completed 2.5 turns. The width of the insulation was 1.4 mm and the thickness was 0.9 mm. The empty space in all models was filled with an ambient medium with conductance varying from saline (2 S/m) to fat (0.04 S/m). For the rat models, ambient mediums were 20 mm in length and 4 mm in diameter. For the rabbit models, they were 120 mm in length and 40 mm in diameter. The outer boundaries of all models were grounded. A 1 mA positive current was applied on one contact, and a 1 mA negative current on the other. Due to the model being purely resistive, the voltage field only needed to be solved for a single current amplitude. Electrical properties for each material were based on field standards and can be found in S1 Table [23–26].

Once the Comsol model was solved, the voltage distribution inside the fascicle was exported and read into a NEURON model consisting of 500 parallel axons uniformly distributed throughout the fascicle. The multi-fascicle nerve had 100 axons in each of the five fascicles. Axons were designed using the model created by McIntyre, Richardson, and Grill [27]. All electrical parameters were identical in this study, but the geometric parameters were interpolated using either a 1^st^ or 2^nd^ order polynomial. All fitted functions can be found in S2 Table. Each fiber was set to the length of the corresponding Comsol model, either 20 mm or 120 mm. Diameters were taken from a normal distribution meant to represent A-fibers (rat sciatic: 6.87 ± 3.02 μm, rat vagus: 2.5 ± 0.75 μm, rabbit sciatic: 8.85 ± 3.1 μm) [28,29]. Rat vagus fiber diameters were estimated based on the conduction velocity of the fibers mediating the Hering-Breuer reflex [30–33]. In both sciatic models, a lower cutoff of 2 μm diameter was used. In the vagus model, a cutoff of 1 μm was used.

After a 0.5 ms delay to ensure all axons had reached a steady baseline, a biphasic pulse of varying current amplitude was applied to the NEURON model. The voltage field calculated in Comsol was linearly scaled to the specified current and applied for 0.1 ms, and then the inverse was applied immediately after for another 0.1 ms. Voltage traces from nodes at the proximal end of the axon were recorded and used to determine whether that axon was activated at the given current amplitude. The activation data was used to create dose-response curves showing the percentage of axons activated as a function of current amplitude.

### Animal experiments

All handling, housing, stimulation, and surgical procedures were approved by The University of Texas at Dallas Institutional Animal Care and Use Committee. Twelve Sprague Dawley female rats (Charles River, 3 to 6 months old, 250 to 500 g) were housed in a 12:12 h reverse light-dark cycle. Six rats were used for sciatic experiments, and six rats were used for vagus experiments. Four New Zealand white male rabbits (Charles River, 3 to 6 months old, 2 to 4 kg) were housed in a 12:12 h light-dark cycle. All four rabbits were used for sciatic experiments.

### Electrodes

Rat experiments were performed using custom-made cuff electrodes. All cuff electrodes were hand-made according to standard procedures [34]. The cuffs were insulated with 3 to 6 mm sections of polyurethane tubing with an inner diameter of 1 mm and outer diameter of 2 mm. Electrodes were multi-stranded platinum-iridium wire with a diameter of 0.01 mm. For the circumferential cuff electrode, platinum-iridium wires covered a 270° arc inside the cuff. To approximate a flat electrode, partial contacts were used which only covered a 60° arc. Additionally, an intermediate electrode was tested with a 120° arc. All electrode impedances were measured in saline before testing to ensure proper construction.

Rabbit experiments were performed using both custom-made circumferential electrodes and manufactured flat electrodes. The circumferential electrodes were made using the same materials and protocol as the rat cuff electrodes but sized to accommodate the larger rabbit sciatic nerve (3 mm inner diameter, 4.5 mm outer diameter, 270° arc). Flat electrodes consisted of PCBs connected to two rectangular platinum contacts [35]. All on-board components were encapsulated and hermetically sealed in glass. Current controlled stimulation was delivered with this device using an on-board microcontroller with a digital to analog converter (DAC). Analog output from the DAC was amplified by an op-amp with a maximum current of 1.2 mA. The rectangular contacts were attached to the surface of the glass and connected to the PCB through hermetic through glass vias. A 9-turn 3-layer coil was used as an antenna for power reception and communication. A silicone sleeve was fitted around the device to serve as an insulating cuff.

### Rat sciatic nerve stimulation

Rats were anesthetized using ketamine hydrochloride (80 mg/kg, intraperitoneal (IP) injection) and xylazine (10 mg/kg, IP) and given supplemental doses as needed. Once the surgical site was shaved, an incision was made on the skin directly above the biceps femoris [15,36]. The sciatic nerve was exposed by dissecting under the biceps femoris. The gastrocnemius muscle was separated from skin and surrounding tissue. Cuff electrodes were then placed on the sciatic nerve with leads connected to an isolated programmable stimulator (Model 4100; A-M Systems^™^; Sequim, WA). The nerve was left in place underneath the biceps femoris and the cavity was kept full of saline at all times to ensure that the cuff would be operating in a uniform medium with conductance similar to tissue. The Achilles tendon was severed at the ankle and affixed to a force transducer using nylon sutures. The foot was clamped and secured to a stereotaxic frame to prevent movement of the leg during stimulation and to isolate recordings from the gastrocnemius muscle.

Stimulation was delivered through the A-M Systems^™^ Model 4100. Voltage traces were recorded using a digital oscilloscope (PicoScope® 2204A; Pico Technology; Tyler, TX). The force of muscle contraction was recorded through a force transducer (2kg EBB Load Cell; Transducer Techniques; Temecula, CA) which was connected to an analog channel on an Arduino® Mega 2560. All components were integrated using MATLAB®. Data was sampled at 10 Hz.

Stimulation consisted of 0.5 second trains of biphasic pulses (100 μs pulse width) at 30 Hz with varying current amplitudes ranging from 20–800 μA. Stimulation intensities were randomly interleaved. Values for current were manually set in each experiment to ensure that the range of values included the entire dose-response curve. Stimulation was delivered every 15 seconds and each parameter was repeated in triplicate.

### Rat Vagus nerve stimulation

Rats were anesthetized using ketamine hydrochloride (80 mg/kg, IP) and xylazine (10 mg/kg, IP) and given supplemental doses as needed. An incision and blunt dissection of the muscles in the neck exposed the left cervical vagus nerve, according to standard procedures [37–39]. The nerve was placed into the cuff electrode, and leads from the electrode were connected to the programmable stimulator. The cavity was kept full of saline at all times. To assess activation of the vagus nerve, blood oxygen saturation (SpO2) was recorded using a pulse-oximeter (Starr Life Sciences^™^, MouseOx Plus®) as previously described [31]. Data was read into MATLAB® using a Starr Link Plus^™^ with the outputs connected to analog channels on the Arduino®. Data was sampled at 10 Hz and filtered with a 10 sample moving average filter.

Stimulation consisted of 5 second trains of biphasic pulses (100 μs pulse width) at 30 Hz with varying current amplitudes ranging from 50–2500 μA. Values for current were randomly interleaved. Stimulation was delivered every 60 seconds, but was delayed if needed to allow the oxygen saturation to return to baseline. Each parameter was repeated twice.

### Rabbit sciatic nerve stimulation

Both hind legs of the rabbit were shaved over the incision site the day before surgery. Anesthesia was induced with 3% inhaled isoflurane at 3 L/min. A single intraperitoneal injection of ketamine hydrochloride (35 mg/kg) and xylazine (5 mg/kg) was given after induction. Isoflurane was maintained throughout the experiment. Eye ointment was applied to both eyes to prevent drying. Rectal temperature and breathing were monitored throughout the procedure. The incision sites were cleaned with 70% ethanol, followed by povidone-iodine, followed again by 70% ethanol. An incision site was made along the axis of the femur. The sciatic nerve was exposed with blunt dissection to separate the biceps femoris and quadriceps femoris muscles. Alm retractors were placed to allow cuff implantation. After placing the cuff around the nerve, the retractors were withdrawn.

Stimulation consisted of 0.5 second trains of biphasic pulses (100 μs pulse width) at 10 Hz with varying current amplitudes ranging from 20–1600 μA. Stimulation using the circumferential cuff electrode was delivered using the same system described above for the rat sciatic. Stimulation with the glass-encapsulated electrode was delivered directly from the PCB. The on board stimulation circuit had a resolution of 33μA, which was too large to accurately fit sigmoid functions to the fiber recruitment curve in most cases. Values for current were randomly interleaved. Stimulation was delivered every 5 seconds and each parameter was repeated in triplicate. Data was sampled at 500 Hz using the same load cell collection system described above.

### Analysis and statistical comparisons

**Fig 1:**
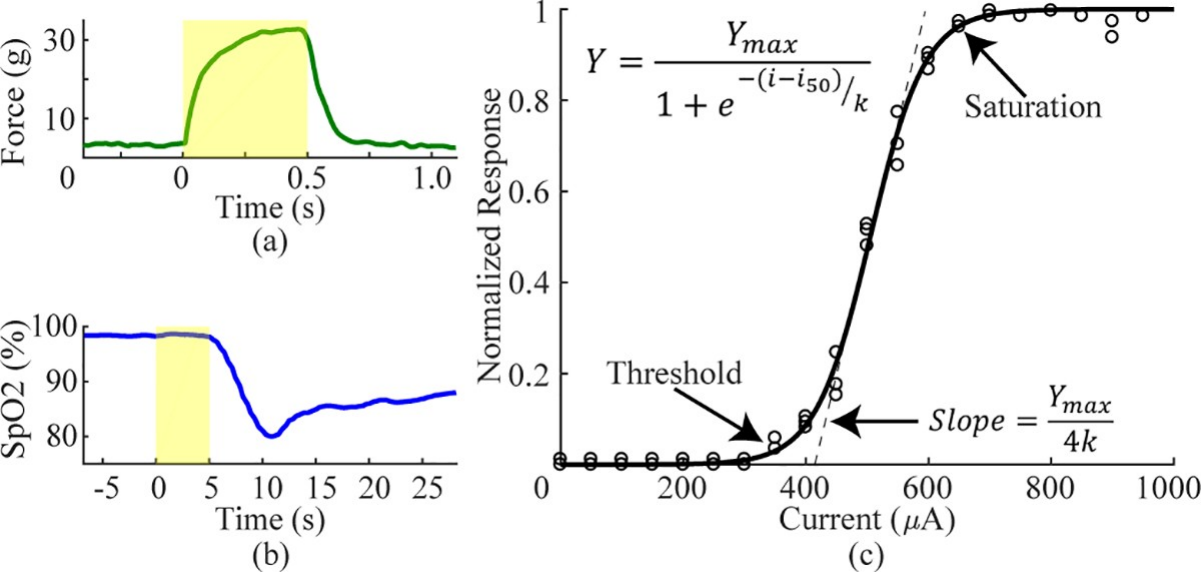
Analysis of fiber recruitment. a) To assess sciatic nerve recruitment, we measured force of hindlimb muscle contraction in response to a range of stimulation intensities. Shaded region represents stimulation at 30Hz for 0.5 seconds. b) To measure vagus nerve recruitment, we measured reductions in SpO2. Shaded region represents stimulation at 30Hz for 5 seconds. c) An example recruitment curve with a fitted sigmoid function and all outcome measures identified.

All responses were normalized to the maximum response recorded in each subject. As the maximal value recorded in the same subject with one electrode may be lower than the maximal value with a different electrode due to the small expected variance across preparations, the average recruitment does not reach 100%. Raw, non-normalized responses are included in the supplementary information (S4, S5 and S6 Figs). Dose-response curves were fitted with a sigmoid function (Fig 1C). Restrictions were placed on the fitted curve such that the point at 1% of Y_max_ could not be at a negative current intensity. For each curve, the slope was calculated at the midpoint of the fitted function. The threshold was determined by finding the lowest current amplitude that always resulted in a change in the signal of force or SpO2 greater than 3x the standard deviation of the preceding 1 second of signal for muscle activation or 10 seconds of signal for SpO2. The saturation point was determined by finding the lowest current value that produced a change in the signal greater than 90% of the mean of the top 50% of the curve. The dynamic range was calculated as the saturation point minus the current value one step below the threshold. All analyses were verified by a blinded experimenter.

Data reported in the text and figures represent mean ± standard error of the mean (SEM). The sample size shown in each figure is equal to the total number of experiments performed with each electrode design, not the number of animals. Thresholds, saturation points, dynamic ranges, and slope of each electrode design were compared on the rat sciatic using a one-way ANOVA after confirming equal variance with a Bartlett test. Individual comparisons were made using post-hoc Tukey-Kramer tests. For the rat vagus and rabbit sciatic, the variance of each metric was compared with a two-sample F-test and then the data were compared using a two tailed two-sample t-test with either equal or unequal variance depending on the F-test. The statistical test used for each comparison is noted in the text. All calculations were performed in MATLAB.

## Results

### One-sided and circumferential electrodes provide equivalent recruitment of rat sciatic nerve

Flat electrode contacts that do not surround the entire nerve may require more current to activate the whole nerve than circumferential electrode contacts. We measured recruitment using computational modeling and *in vivo* experiments on the rat sciatic nerve. To represent flat electrodes, we used a modified circumferential electrode that only provided 60° of coverage compared to the standard 270°. Fiber recruitment functions were created using the 60° and 270° designs as well as an intermediate 120° design.

#### Model

**Fig 2:**
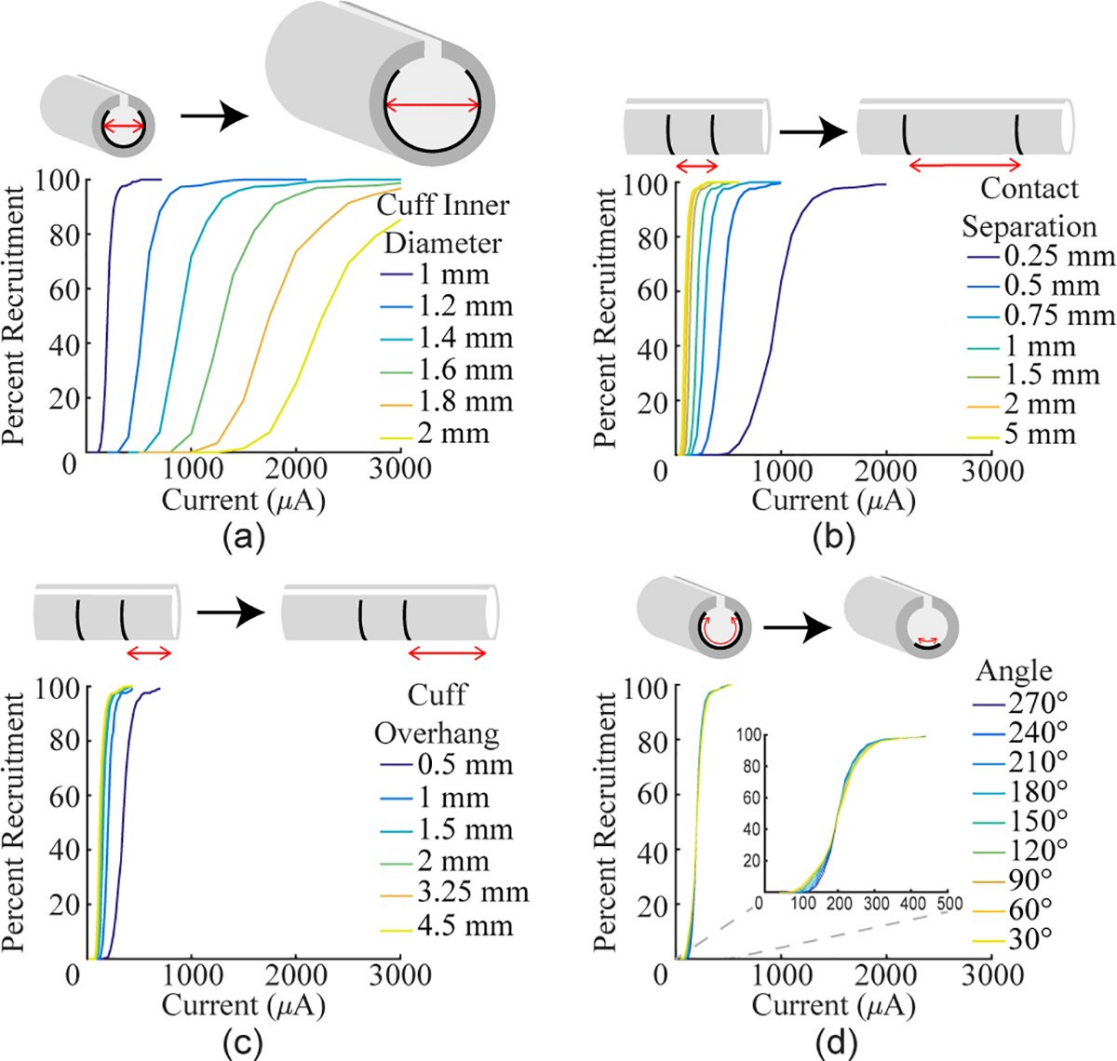
Modeling the effect of various cuff electrode parameters on recruitment of rat sciatic nerve. Recruitment curves were generated with different values for several design parameters. a) Increasing the inner diameter of the cuff (1 mm contact separation, 1 mm cuff overhang, 270°) drastically reduces recruitment. b) Increasing the distance between the two stimulating contacts (1 mm cuff inner diameter, 1 mm cuff overhang, 270°) increases recruitment. c) Increasing the amount of cuff overhang (1 mm cuff inner diameter, 1 mm contact separation, 270°) increases recruitment. d) Reducing the angle of coverage (1 mm cuff inner diameter, 1 mm contact separation, 1 mm cuff overhang) has minimal effect on recruitment.

We used computational modeling to evaluate fiber recruitment using multiple electrode designs with different values for angle of coverage, contact spacing, cuff overhang, and cuff inner diameter. Reducing the angle of coverage had a small effect on recruitment (Fig 2D). The smallest angle (30°) required 105.2 μA to recruit 5% of fibers (*i*_5%_) whereas the standard angle (270°) required 143.4 μA. To recruit 95% of fibers (*i*_95%_), the smallest angle required 311.7 μA and the standard angle required 296.0 μA.

Unlike angle of coverage, the other three variables strongly influenced recruitment. With a standard 270° arc, increasing the inner diameter of the cuff had the strongest effect on recruitment, greatly increasing both the threshold and saturation current (Fig 2A; 1 mm: *i*_5%_ = 143.4 μA, *i*_95%_ = 296.0 μA; 1.2 mm: *i*_5%_ = 378.6 μA, *i*_95%_ = 807.7 μA). Increasing the distance between the contacts lowered both the threshold and saturation current (Fig 2B; 0.25 mm: *i*_5%_ = 332.3 μA, *i*_95%_ = 697.3 μA; 5 mm: *i*_5%_ = 41.8 μA, *i*_95%_ = 119.2 μA). Increasing the amount of cuff overhang lowered both the threshold and saturation current (Fig 2C; 0.5 mm: *i*_5%_ = 232.3 μA, *i*_95%_ = 490.0 μA; 4.5 mm: *i*_5%_ = 88.4 μA, *i*_95%_ = 202.1 μA). Compared to the impact of the three other variables, angle of coverage was the least important factor, suggesting that it is not a critical factor in electrode design and flat electrodes would achieve saturation at similar current amplitudes to circumferential electrodes. Varying the cuff inner diameter, contact separation, and cuff overhang on a 60° electrode demonstrated that each variable affects an electrode with a shorter arc similar to a standard electrode (S1 Fig).

#### Empirical

**Fig 3:**
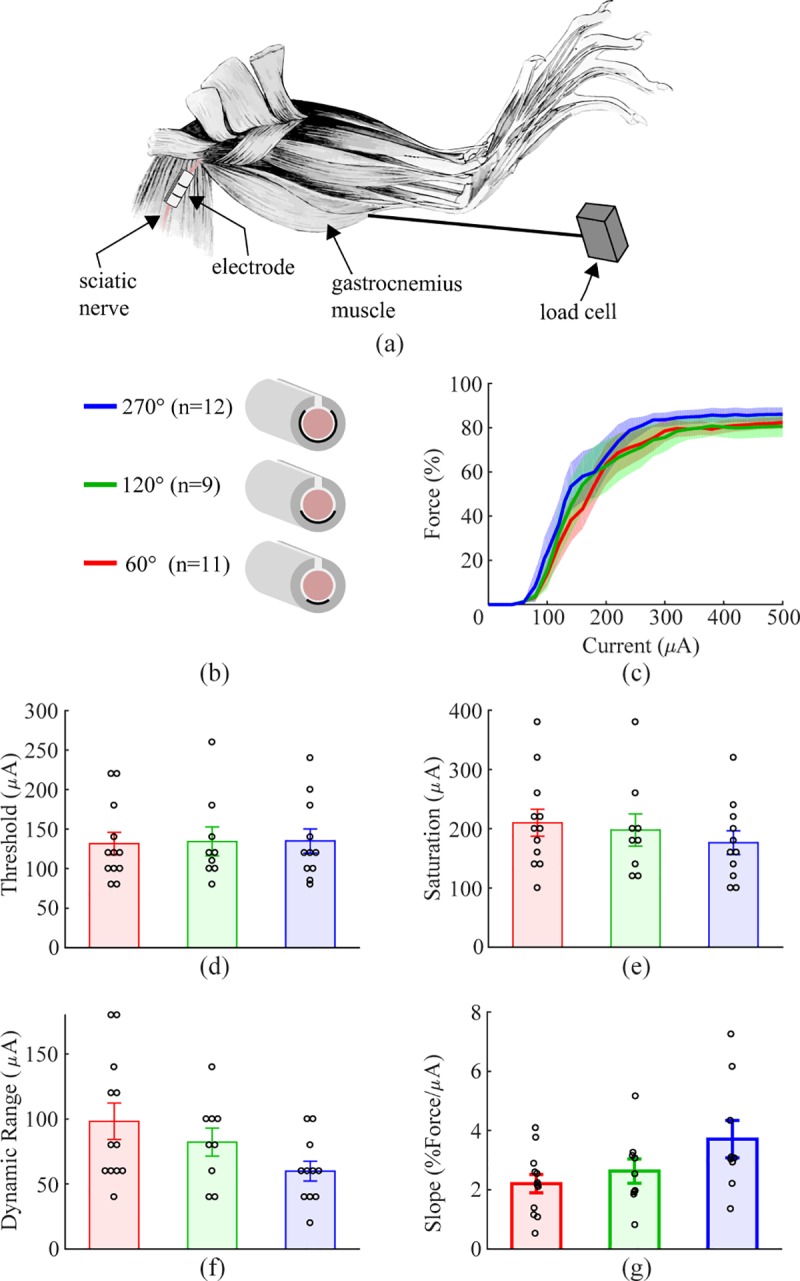
Approximately flat electrode does not reduce fiber recruitment in rat sciatic nerve. a) Schematic diagram of the experimental setup. b) Schematic diagram of the three cuff electrode designs tested on the rat sciatic nerve. c) Force generated as a function of stimulation intensity for each electrode design. All geometries result in similar recruitment. Shaded regions represent SEM. d-g) Thresholds, saturation currents, dynamic ranges, and slopes are similar for each electrode design. Data indicate mean ± SEM, and circles represent individual data.

To confirm modeling predictions, we evaluated nerve recruitment in the rat sciatic nerve using the 60°, 120°, and 270° electrodes. In vivo data closely resembled data derived from the model, with flat and circumferential contacts demonstrating comparable fiber recruitment. No significant differences were found between recruitment thresholds for any of the electrode configurations (Fig 3D; 60°: 131.7 ± 14.2 μA, 120°: 134.4 ± 18.3 μA, 270°: 135.0 ± 15.4 μA; Bartlett’s test, χ2(0.05, 2) = 0.110, p = 0.94639, one-way ANOVA, F(2, 29) = 0.01, p = 0.986). Additionally, ANOVA did not reveal differences in saturation current, dynamic range, or slope between the electrode designs (Saturation: Fig 3E, 60°: 205.0 ± 23.6 μA, 120°: 195.6 ± 27.2 μA, 270°: 176.4 ± 20.0 μA, Bartlett’s test, χ2(0.05, 2) = 0.448, p = 0.799, one-way ANOVA, F(2, 29) = 0.41, p = 0.569; Dynamic range: Fig 3F, 60°: 93.3 ± 13.6 μA, 120°: 80.0 ± 10.5 μA, 270°: 60.0 ± 7.6 μA, Bartlett’s test, χ2(0.05, 2) = 4.310, p = 0.116, one-way ANOVA, F(2, 29) = 2.41, p = 0.0647; Slope: Fig 3G, 60°: 2.21 ± 0.31%Force/μA, 120°: 2.63 ± 0.41%Force/μA, 270°: 3.71 ± 0.63%Force/μA, Bartlett’s test, χ2(0.05, 2) = 3.195, p = 0.202, one-way ANOVA, F(2, 29) = 3.03, p = 0.065). These results confirm that the one-sided electrodes and circumferential electrodes yield equivalent nerve recruitment across a range of stimulation intensities.

### One-sided electrodes recruit more efficiently than circumferential electrodes in the rat vagus nerve

We next tested recruitment using the same 60° and 270° cuff electrodes on the rat vagus nerve, which has a smaller diameter and different fascicular organization.

#### Model

**Fig 4:**
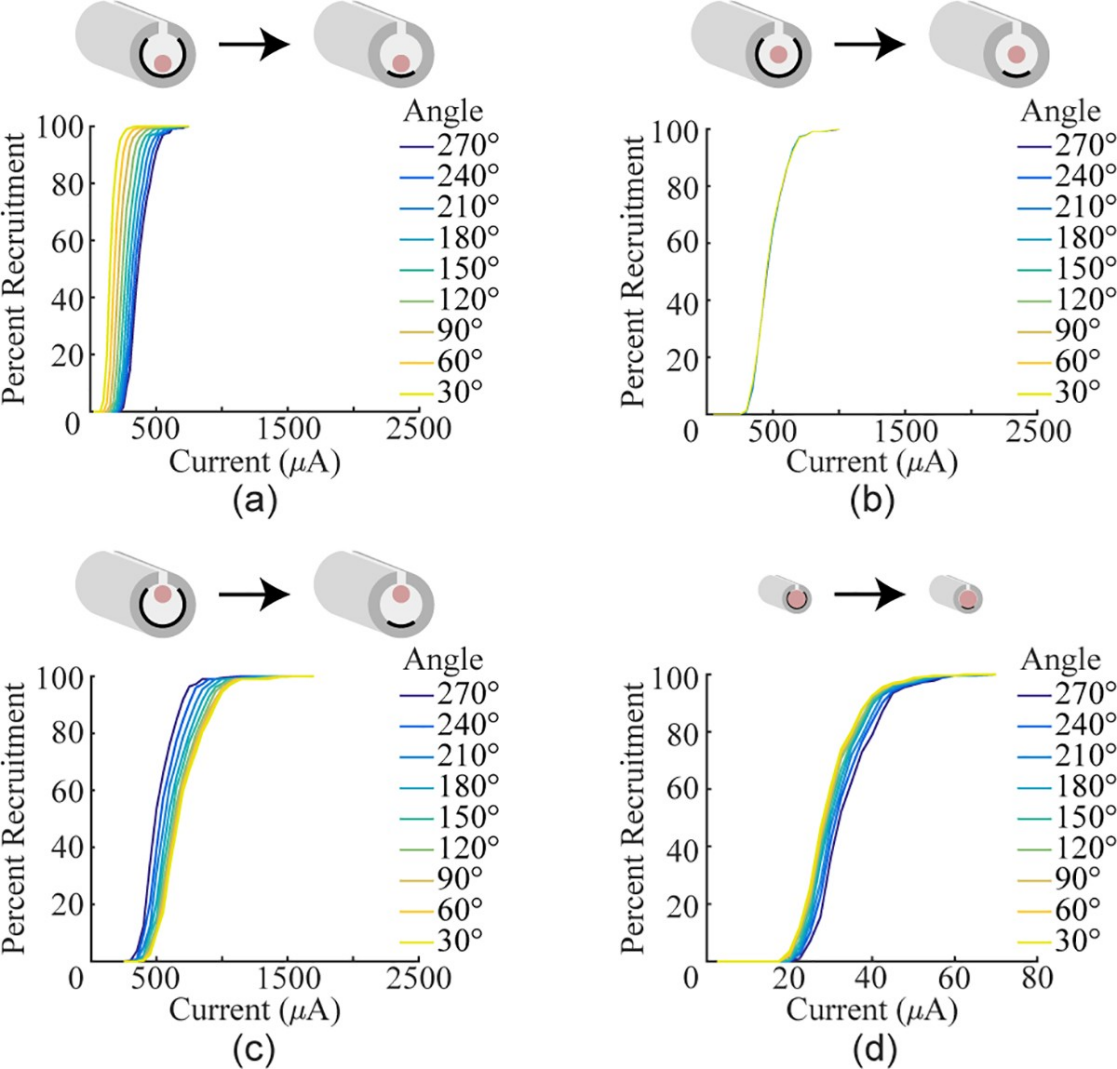
Reducing angle of coverage increases fiber recruitment in a model of the rat vagus nerve. a) Recruitment curves generated using a cuff with a 1 mm inner diameter, but with the nerve positioned at next to the contacts. Reducing the angle increases recruitment. b) Recruitment curves generated using a cuff with a 1 mm inner diameter, but with the nerve positioned in the middle of the cuff lumen. Reducing the angle has no effect. c) Recruitment curves generated using a cuff with a 1 mm inner diameter, but with the nerve on the opposite side of the cuff lumen from the contacts. Reducing the angle decreases recruitment. d) Recruitment curves generated by modeling cuff electrodes with various angles of completion around the rat vagus. Instead of a 1 mm inner diameter, the cuff diameter was set to 0.44 mm to keep the ratio of the cuff diameter to nerve the same as in the sciatic model. When the cuff is sized to fit the nerve, reducing the angle has little effect on fiber recruitment.

Modeling of the rat vagus showed an unexpected result: decreasing the angle of the electrodes improved recruitment by decreasing both the threshold and saturation current (Fig 4A; 30°: *i*_5%_ = 101.6 μA, *i*_95%_ = 224.3 μA; 270°: *i*_5%_ = 262.0 μA, *i*_95%_ = 532.4 μA). To explain this result, three follow up tests were run. In the original model, the vagus nerve was positioned at the bottom of the cuff lumen very close to the contacts to match the experimental prep. The first two follow up tests changed the nerve’s position to be either in the middle of the cuff or on the opposite side from the contacts. When the nerve was in the middle of the cuff, changing the angle of coverage had no effect on recruitment (Fig 4B; 30°: *i*_5%_ = 315.9 μA, *i*_95%_ = 679.4 μA; 270°: *i*_5%_ = 324.3 μA, *i*_95%_ = 674.0 μA). When the nerve was on the opposite side of the cuff, reducing angle of coverage increased both the threshold and saturation current (Fig 4C; 30°: *i*_5%_ = 467.4 μA, *i*_95%_ = 1013.9 μA; 270°: *i*_5%_ = 357.9 μA, *i*_95%_ = 735.5 μA). The final follow up test varied the angle of coverage inside of a cuff which was properly sized for the rat vagus (0.44 mm inner diameter). In this case, varying the angle of coverage once again had minimal effect on recruitment (Fig 4D; 30°: *i*_5%_ = 20.5 μA, *i*_95%_ = 42.0 μA; 270°: *i*_5%_ = 24.2 μA, *i*_95%_ = 47.2 μA). These data suggest that in a cuff that is significantly larger than the nerve, the vagus benefits from the increased current density near the contacts present with shorter angles of coverage without being affected by the decreased current density far from the contacts.

#### Empirical

**Fig 5:**
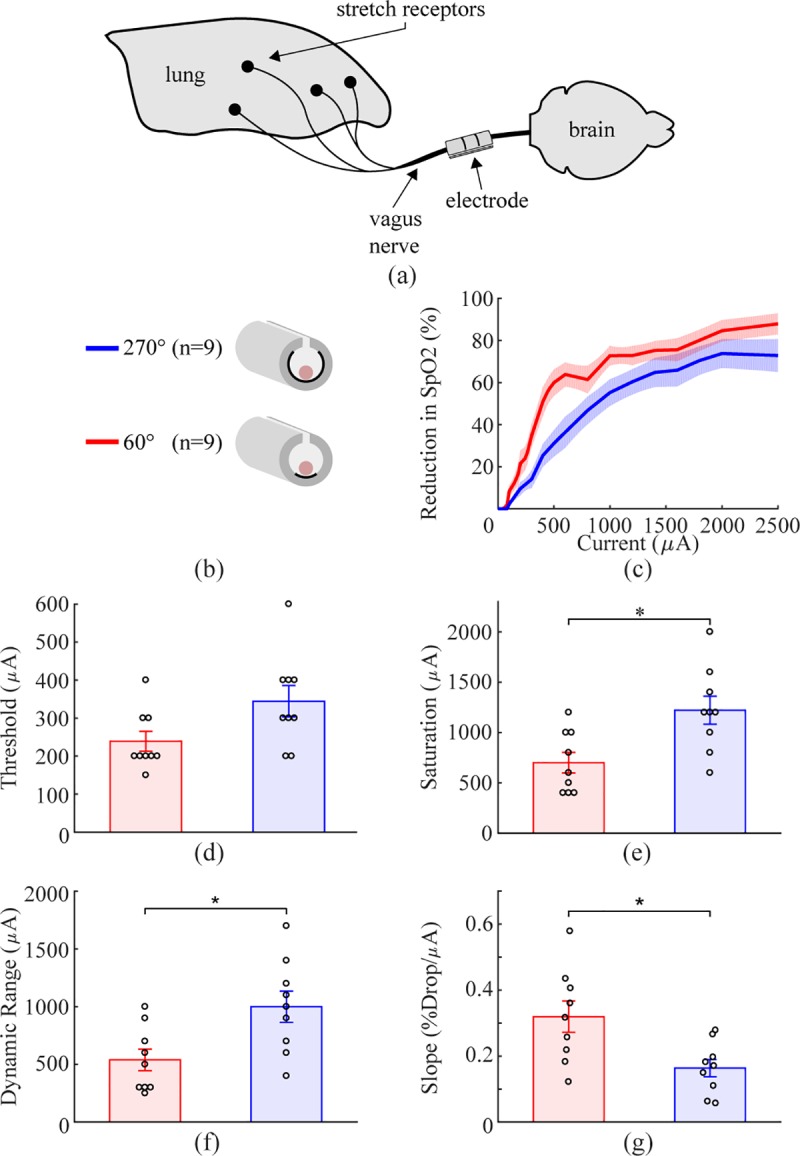
Reducing angle of coverage to approximate a flat electrode increases fiber recruitment in rat vagus nerve. a) Schematic diagram of the experimental setup. b) Schematic diagram of the two cuff electrode designs tested on the rat vagus nerve. c) Decreases in SpO2, a biomarker of vagal activation, as a function of stimulation intensity for each electrode design (y-axis is percent of maximum reduction). Similar to modeling results, the decreased angle of coverage generates more efficient nerve recruitment. d-g) Thresholds are similar for each design. The 60° electrodes displayed reduced saturation current, dynamic range, and increased slope compared to the 270° electrodes. Data indicate mean ± SEM, and circles represent individual data.

We next sought to confirm these findings *in vivo*. To evaluate activation of vagus nerve fibers, we measured rapid stimulation-dependent reduction in oxygen saturation, a well-described biomarker of vagus nerve stimulation ascribed to activation of the Hering-Breuer reflex [31]. Stimulation of vagal A-fibers, including the pulmonary stretch receptors, temporarily prevents inhalation and causes blood oxygen saturation to fall (Fig 5A) [30]. As a result, measurement of oxygen saturation provides a simple means to assess vagal A-fiber recruitment.

The 60° electrodes recruited fibers more effectively than the 270° electrodes, corroborating findings from the model. A trend toward reduced threshold was observed with the 60° electrode, although this failed to achieve statistical significance (Fig 5D; 60°: 238.9 ± 26.1 μA, 270°: 344.4 ± 41.2 μA; two tailed paired t-test, p = 0.0508). The 60° electrode displayed a significantly reduced saturation current, dynamic range, and increased slope compared to the 270° electrode (Saturation: Fig 5E, 60°: 700 ± 102.7 μA, 270°: 1222 ± 139.2 μA, two tailed paired t-test, p = 8.5x10^-3^; Dynamic Range: Fig 5F, 60°: 538.9 ± 93.5 μA, 270°: 1000 ± 135.4 μA, two tailed paired t-test, p = 0.014; Slope: Fig 5G, 60°: 0.320 ± 0.047, 270°: 0.164 ± 0.026, two tailed paired t-test, p = 0.0165). Both the model and empirical data demonstrate that the one-sided electrodes have a steeper recruitment curve and lower saturation current than circumferential electrodes.

### Flat and circumferential electrodes provide equivalent recruitment in rabbit sciatic nerve

The results presented above support the notion that flat electrodes provide at least as effective fiber recruitment as circumferential electrodes. However, whereas the 60° electrodes used in the above experiments contact only a single side of the nerve similar to a flat electrode, they are not truly flat and thus do not capture all the features of the geometry that may influence fiber recruitment. Therefore, we sought to confirm these results using a true flat electrode. The electrode was manufactured on a printed circuit board (PCB), encapsulated in glass, and inserted into a silicone sleeve that acted as an insulating cuff. These electrodes were tested on the rabbit sciatic nerve, which is an order of magnitude larger than the rat sciatic nerve [15,17].

#### Model

**Fig 6:**
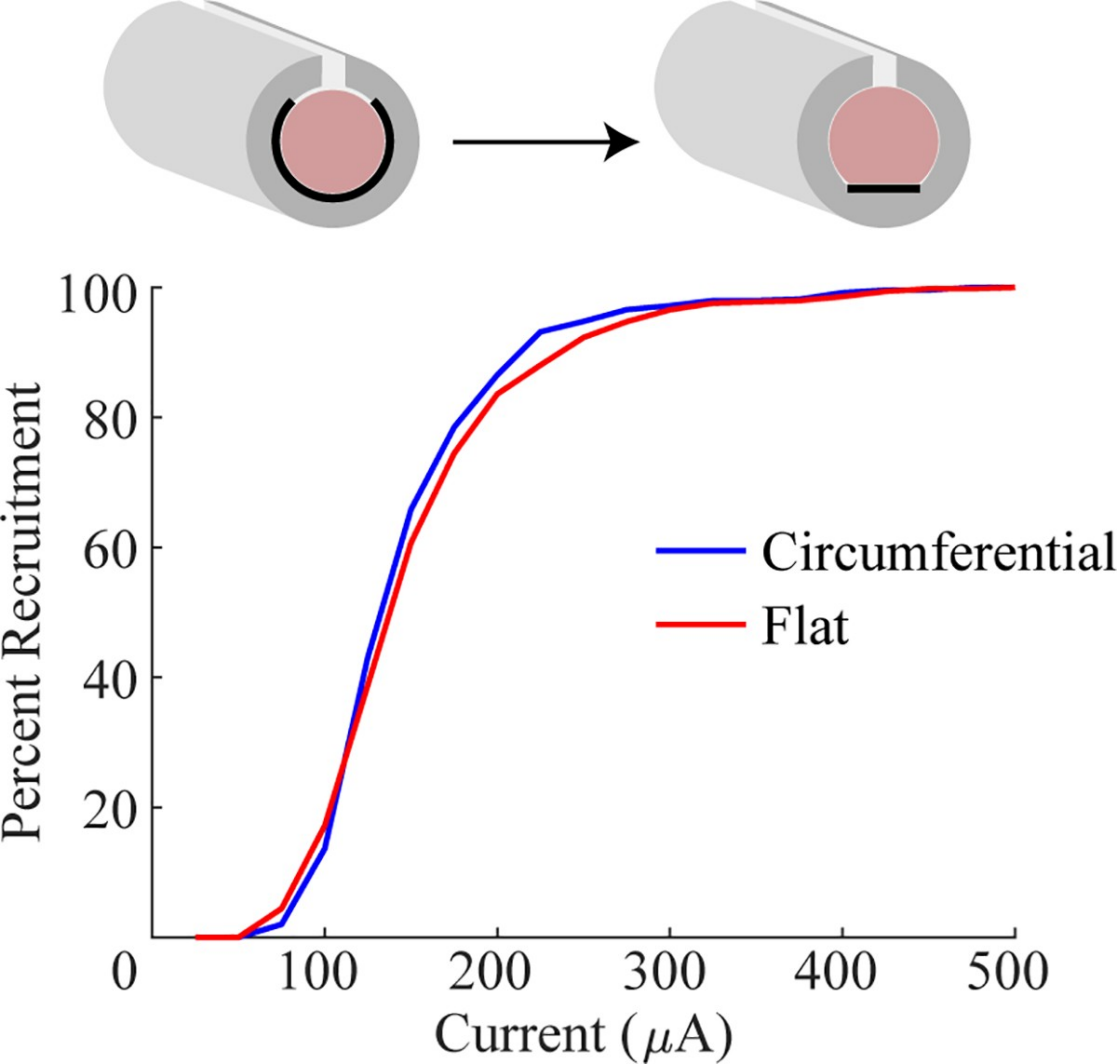
Flat and circumferential electrodes provide similar recruitment in a model of the rabbit sciatic nerve. Recruitment curves generated by modeling cuff electrodes around the rabbit sciatic nerve with either flat or circumferential contacts. Note the similarity in fiber recruitment.

**Fig 7:**
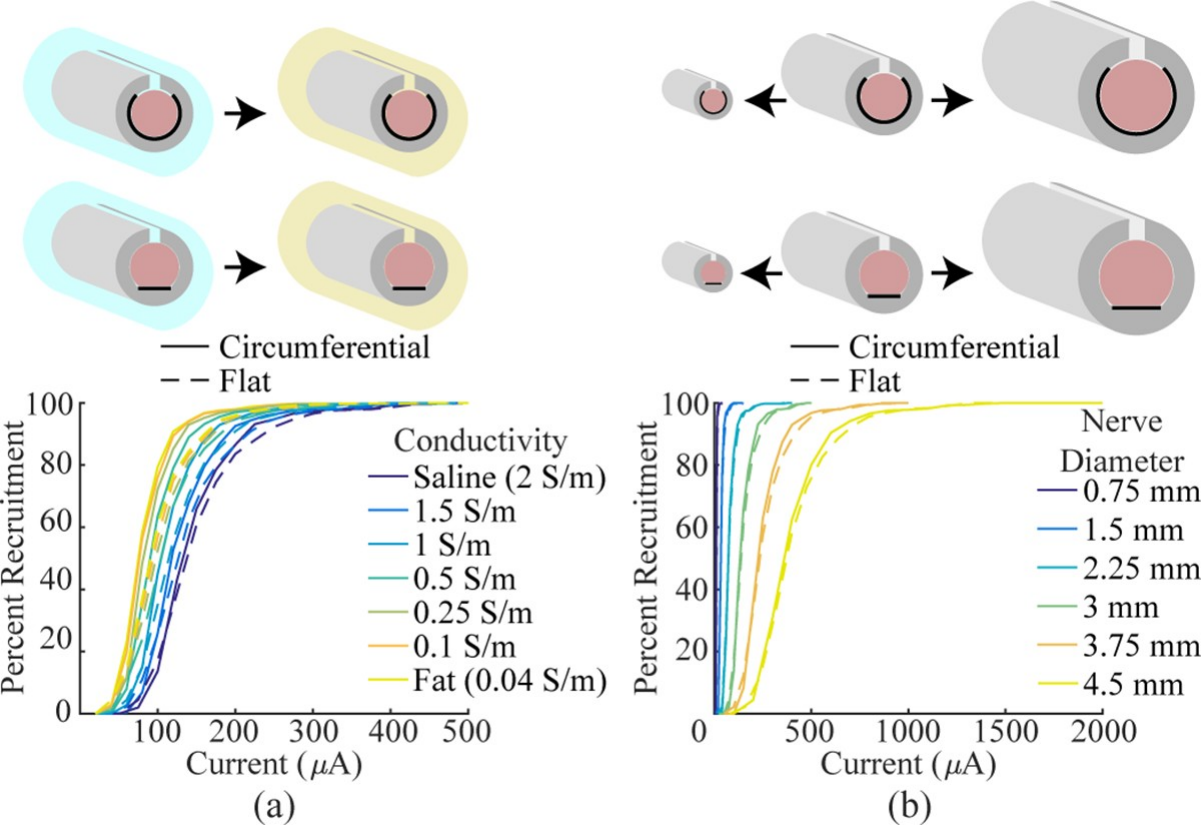
Models of flat and circumferential electrodes in various extracellular media and on various nerve sizes. a) Recruitment curves generated by modeling flat and circumferential electrodes in various ambient mediums. The conductivity of the ambient medium was varied from saline to fat. As expected, the extracellular medium influences recruitment efficiency, but recruitment is similar between the two electrode designs in all cases. b) Recruitment curves generated by modeling flat and circumferential electrodes on various diameter nerves. All features of the cuff electrode were kept proportional and scaled to match the nerve. In all cases, recruitment is similar between the two designs.

We performed modeling to evaluate recruitment using flat contacts and circumferential contacts. The cross-sectional area of the nerve and of the cuff lumen was matched between the circumferential and flat electrode models by increasing the inner diameter of the flat cuff by 8.67% and modifying the nerve shape to fit (Fig 6) [22]. Flat and circumferential designs had similar thresholds and saturation currents (Fig 6; Flat: *i*_5%_ = 76.1 μA, *i*_95%_ = 278.6 μA; Circumferential: *i*_5%_ = 81.4 μA, *i*_95%_ = 253.1 μA). Next, flat and circumferential electrodes were compared in different ambient mediums with conductivities ranging from fat to saline. Decreasing the conductivity of the ambient medium increased recruitment, but the comparable recruitment observed with flat and circumferential contacts was consistent in all cases (Fig 7A). Flat and circumferential electrodes were compared on nerves of varying size by increasing or decreasing the spatial scale of the original rabbit sciatic model. Larger nerves required more current to achieve similar levels of fiber recruitment, but once again, flat and circumferential electrodes were similar in all cases (Fig 7B).

**Fig 8:**
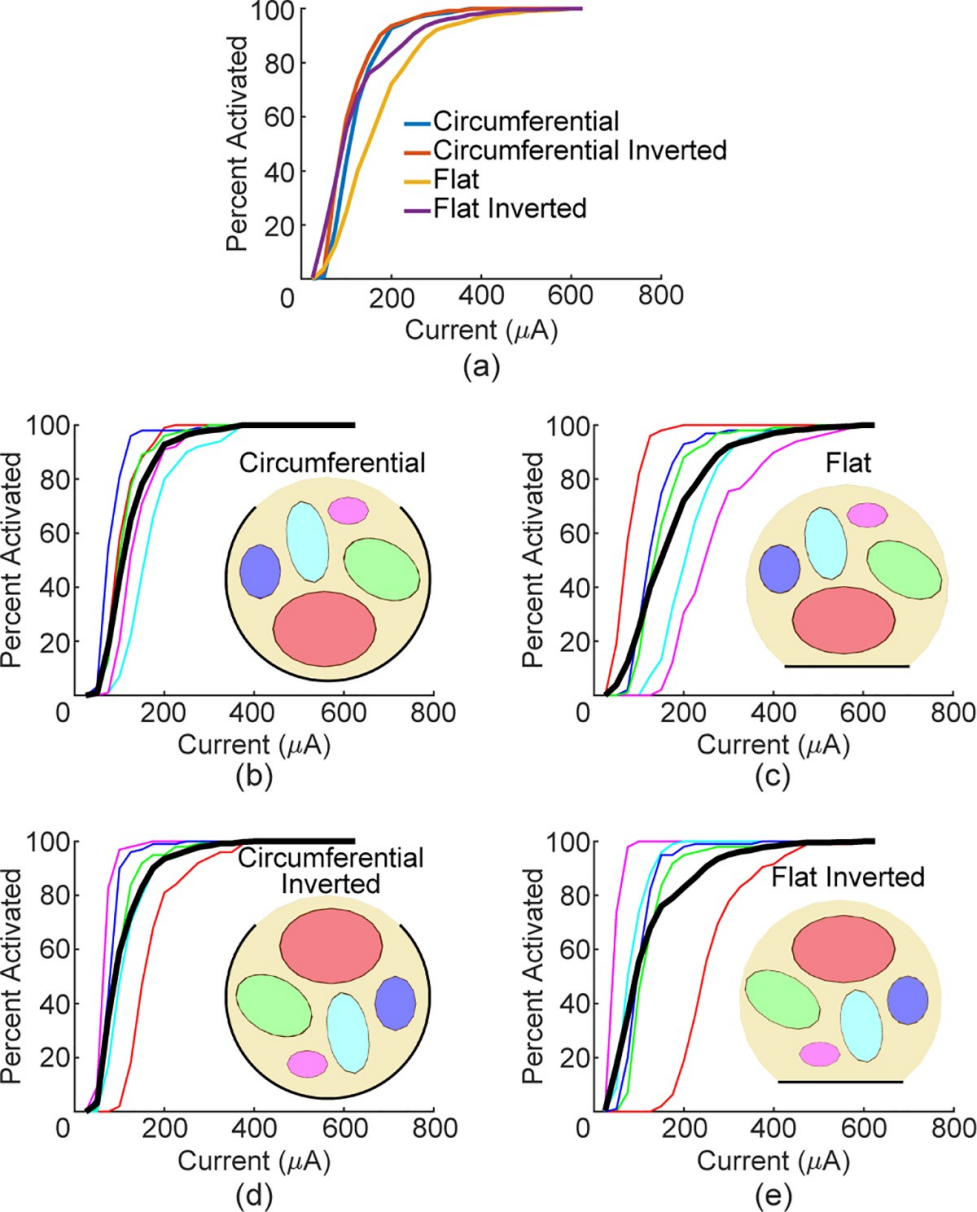
Flat electrodes result in greater threshold variability for individual fascicles, but similar recruitment of the whole nerve. a) Whole nerve recruitment curves for the four combinations modeled. b-e) Recruitment curves for each fascicle (line color corresponds to fascicle of same color) and whole nerve recruitment (thick black line).

For comparison to commonly used clinical VNS electrodes, we modeled a helical electrode design around the nerve. Recruitment was similar to the flat electrode design (S2 Fig). In humans, the vagus nerve is comprised of multiple fascicles [40]. To reflect this, we modeled flat and circumferential electrodes on a nerve containing five fascicles. Recruitment of the nerve as a whole was similar between the two designs despite each fascicle being recruited differently. The variance in thresholds for activation of each fascicle was greater with the flat design (Fig 8). These data suggest that flat electrodes recruit the nerve as a whole similarly to currently used electrode designs even though recruitment of individual fascicles may differ.

#### Empirical

**Fig 9:**
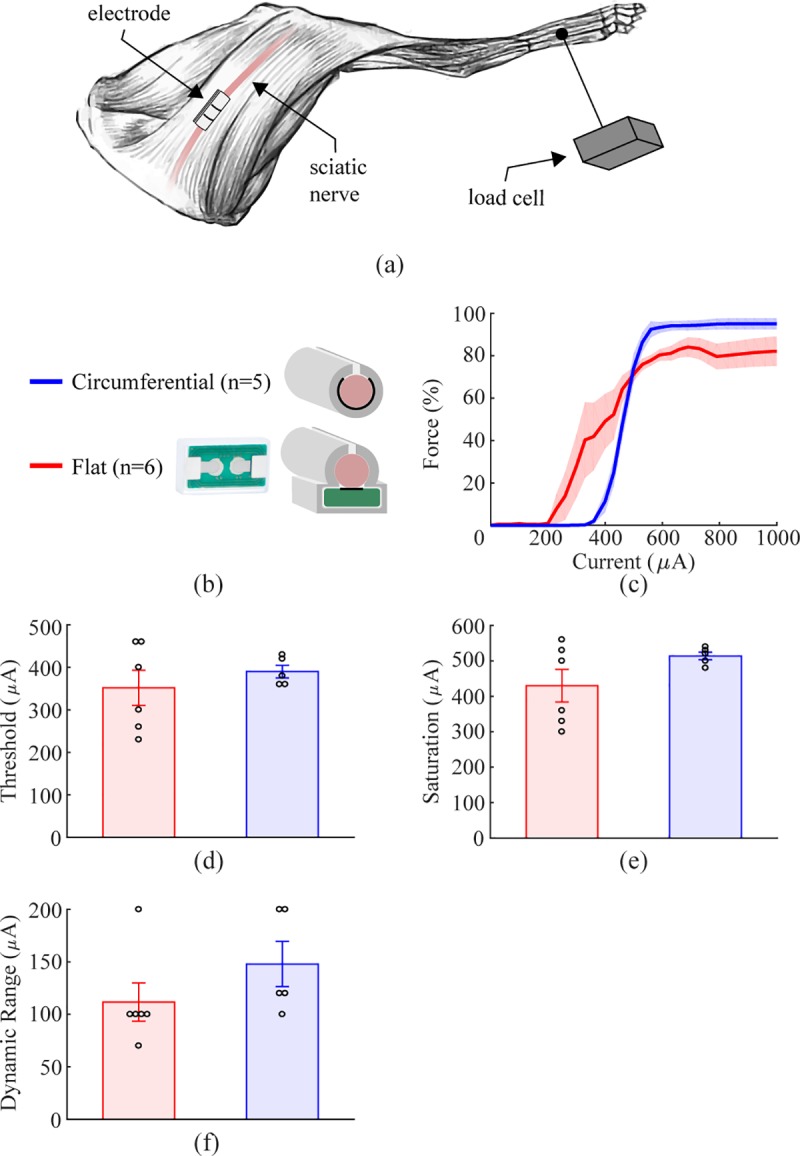
Flat and circumferential electrodes provide similar recruitment of rabbit sciatic nerve. a) Schematic diagram of the experimental setup. b) Schematic diagram of the two cuff electrode designs tested on the rabbit sciatic nerve. c) Force generated as a function of stimulation intensity for flat and circumferential electrodes. Both designs achieve efficient recruitment of the sciatic nerve, consistent with modeling predictions. d-f) Thresholds, saturation current, and dynamic range are similar for each electrode design. Data indicate mean ± SEM, and circles represent individual data.

To confirm modeling predictions, we evaluated nerve recruitment in the rabbit sciatic nerve by measuring the force of muscle contraction. No difference was found between the thresholds, saturation currents, or dynamic ranges (Threshold: Fig 9D, Circumferential: 390.0 ± 14.8 μA, Flat: 351 ± 41.5 μA, two-sample F-test, F(5, 4) = 9.397, p = 0.0497, two tailed two-sample t-test with unequal variance: p = 0.4167; Saturation: Fig 9E, Circumferential: 514.0 ± 10.8 μA, Flat: 430.0 ± 46.0 μA, two-sample F-test, F(5, 4) = 21.931, p = 0.011, two tailed two-sample t-test with unequal variance: p = 0.1301; Dynamic Range: Fig 9F, Circumferential: 148.0 ± 21.5 μA, Flat: 111.7 ± 18.3 μA, two-sample F-test, F(5, 4) = 0.8693, p = 0.860, two tailed two-sample t-test with equal variance: p = 0.228). The higher variance in the thresholds and saturation currents when using a flat electrode can be explained by the orientation of the nerve relative to the contacts. Fascicles on the opposite side of the nerve from the contacts will have a higher threshold of activation than fascicles near the contacts, as was seen in the multi-fascicle nerve model (Fig 8). However, recruitment of the nerve as a whole is not different between the two designs, which suggests that flat electrodes will not impact the clinical efficacy of VNS.

## Discussion

Circumferential and helical electrodes that surround the majority of the nerve provide uniform stimulation throughout the nerve and yield a steep recruitment curve. Flat electrode contacts could facilitate fabrication, but will produce non-uniform stimulation that may reduce activation of distant low-threshold fibers and increase activation of proximal high-threshold fibers. In this study, we tested if the non-uniformity of the field generated by flat electrodes substantially impacted recruitment. Circumferential electrode contacts were compared to flat electrode contacts on multiple nerves and in multiple species. We find that in all cases tested, recruitment is either equivalent or the flat contacts have a steeper recruitment function and lower saturation current.

On the rat sciatic nerve, both modeling and empirical data demonstrate that the reduced angle of coverage, approximating a flat electrode, provides comparable fiber recruitment to circumferential contacts across a wide range of current intensities. These results suggest that flat electrodes will recruit comparably to circumferential electrodes.

It is plausible that nerve diameter and fascicular organization could differentially affect recruitment with various electrode designs. Unexpectedly, the one-sided electrode contacts provided more efficient recruitment of the rat vagus nerve fibers than the circumferential contacts. There was once again no difference in the thresholds, but the 60° contacts had a steeper recruitment curve and lower saturation current, indicating more efficacious fiber recruitment. The modeling data confirmed these results, which can be ascribed to the relatively small size of the vagus nerve compared to the inner diameter of the insulating cuff. The diameter of the vagus nerve is around 0.4 mm, less than one half that of the sciatic, and thus the nerve occupies a substantially smaller cross-sectional area inside the cuff. Cuffs were always placed such that the vagus was resting at the bottom of the cuff and in the middle of the contacts. Additionally, injection current density was higher with the 60° electrodes given their reduced surface area compared to the 270° electrodes [41]. Due to the small size of the nerve relative to the cuff, its position, and the increased current density near the contacts present with the 60° design, the current density within the nerve was higher with the smaller contact angle. Model results suggest that this is only true when the nerve is at the bottom of the cuff and the cuff is significantly larger than the nerve. If the nerve was moved to the opposite side of the cuff, far away from the contacts, the opposite relationship was seen (Fig 4C), and if the cuff was sized appropriately for the vagus, the 60° contacts did not appear significantly different from the 270° contacts (Fig 4D). Regardless, the modeling and empirical data support the notion that flat electrodes provide at least equivalent fiber recruitment.

Whereas the 60° electrode contacts used in the rat experiments contact only a single side of the nerve similar to flat contacts, it is still possible that a true flat electrode would yield significantly less effective fiber recruitment. Thus, we tested nerve activation in the rabbit sciatic nerve using a true flat electrode manufactured on a PCB and compared recruitment to a standard circumferential electrode. Similar to the rat experiments, modeling and empirical testing revealed no substantial difference in fiber recruitment between the flat and circumferential electrode contacts. These results provide further evidence in an independent replicate that flat contacts stimulate as effectively as circumferential contacts. Furthermore, the devices used in these experiments were simple PCBs, which illustrates the convenience of using flat contacts.

Since all empirical testing in this study used circumferential rather than helical electrodes which are more commonly used in the clinic, it is not initially clear whether the stimulation parameters for flat electrodes would be different from current clinical parameters [42]. We modeled the helical electrode design and compared vagus nerve recruitment to recruitment using a flat electrode design. The helical electrode and flat electrode demonstrated comparable recruitment. The narrow insulating structure used by the helical cuff allows some current to bypass the nerve, which increases the amount of stimulation needed compared to a complete cuff electrode (S2 Fig). The open architecture of the helical cuff is equivalent to having very little cuff overhang, which decreases recruitment compared to a full cuff (Fig 2C).

Many nerve stimulation studies have demonstrated the possibility of using partial contacts, similar to the flat electrodes tested here, to achieve selective stimulation [9,11,12,43]. The increased current density near the electrode allows for individual fascicles to be activated without activation of the rest of the nerve if the stimulation is correctly calibrated. This principle is demonstrated by the higher variance in thresholds present with flat electrodes (Figs 8 and 9). However, activation of the nerve as a whole does not appear to be different between the two designs. Thus, the efficacy of VNS therapies is unlikely to be reduced with the use of flat electrodes.

All modeling and *in vivo* experiments in this study measured A-fiber recruitment, but some applications of VNS rely on B- and C-fibers as well [44]. Although the present data do not provide explicit examination of recruitment of these other fiber types with flat and circumferential electrodes, models of smaller diameter fibers suggest that the increase in fiber threshold would scale proportionally between the two electrode designs. Thus, while more current is required to activate smaller diameter fibers, we predict that the increase in current is likely to be similar comparing flat and circumferential designs. Additionally, there was no distinction between motor and sensory fibers in our experiments. While no difference was seen in stimulation of motor fibers in the rat and rabbit sciatic nerves, stimulation of sensory fibers in the rat vagus was different between the two electrode designs. However, we believe this to be due entirely to electrode geometry and placement, and not inherent differences in fiber types. It is likely that with properly sized cuff electrodes, there is no difference between flat and circumferential electrodes in terms of sensory fiber activation. Further work validating this finding *in vivo* is warranted to compare motor and sensory fibers and determine if flat electrodes are viable for VNS applications that rely on B- and C-fibers.

A major limitation of this study is the absence of empirical testing with chronically implanted electrodes. Many changes occur chronically that could result in reduced efficacy of flat electrodes such as glial scar formation, inflammation, and nerve damage [45]. It is possible that some of these phenomena will affect flat electrodes differently than circumferential electrodes leading to electrode failure. Although chronic implants were not experimentally tested in this study, our modeling studies suggest that flat and circumferential electrodes provide comparable recruitment in a range of physiologically plausible extracellular media, which suggests that scar formation will not affect flat electrodes to a greater extent than it does circumferential electrodes. Future studies are needed to provide a direct empirical evaluation of the chronic efficacy of flat electrodes.

Additionally, there is a lack of data on larger diameter nerves (>3 mm) that would be more comparable to the human vagus. While stimulation of small diameter nerves presented here may not necessarily directly translate to the human vagus, the principles demonstrated in this study are unlikely to differ between the two species. Our modeling studies suggest that flat and circumferential electrodes are equivalent on a range of nerve sizes. Moreover, comparison of recruitment in the rat sciatic and rabbit sciatic suggests that larger nerves require more current to achieve the same level of activation, but in both cases recruitment is comparable between flat and circumferential electrodes.

**Fig 10:**
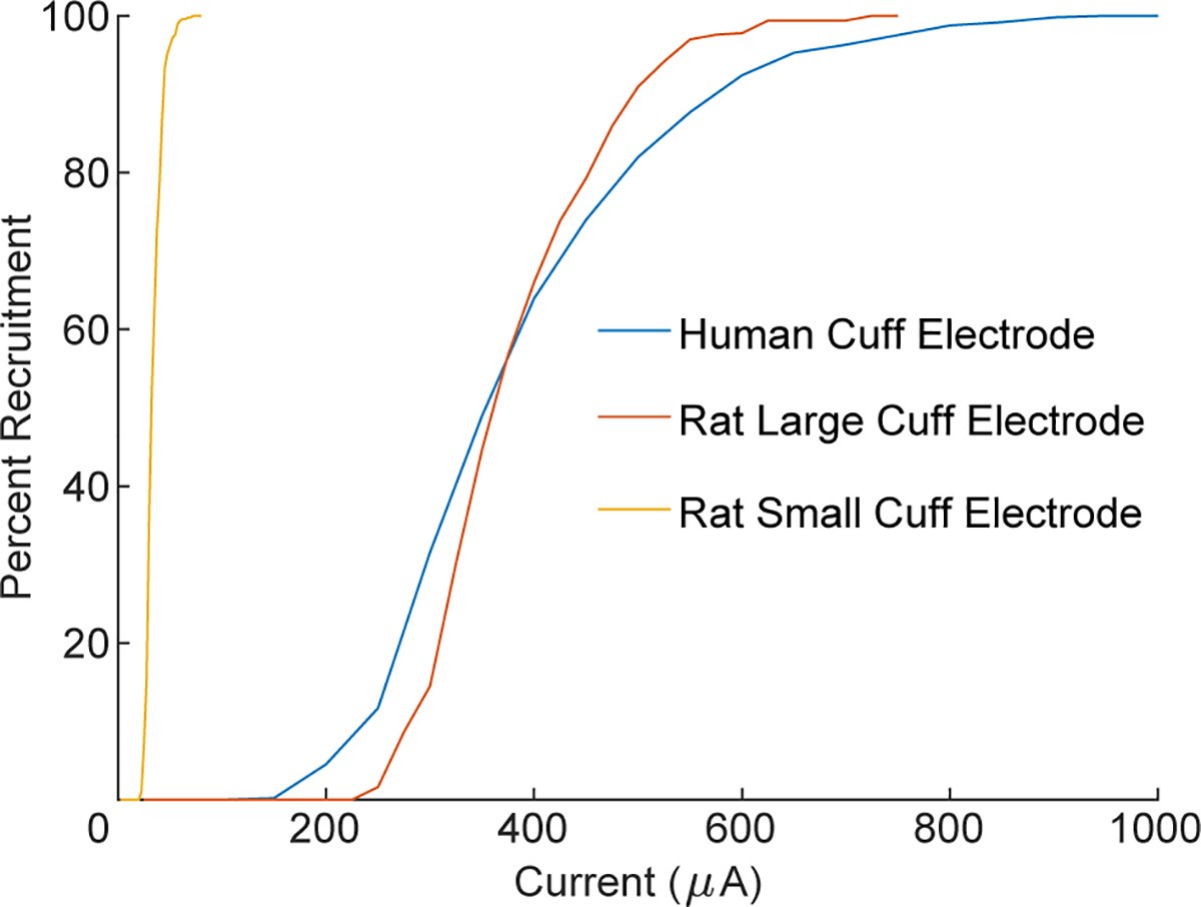
Recruitment of vagus nerve is similar in humans and rats due to cuff electrode design. Larger nerves require more current to recruit, but the therapeutic range of vagus nerve stimulation is similar in rats and humans (Fig 7B). This phenomenon can be explained by the use of tight-fitting stimulating electrodes for human studies and poorly fitting, oversized cuff electrodes for rat studies. Cuff electrodes used in rats are significantly larger than the nerve which leads to inefficient recruitment and brings the two curves into alignment. If rat cuff electrodes were reduced in size, recruitment would be greatly increased. This is consistent with the importance of the ratio of cuff inner diameter to nerve diameter (Fig 2A).

Our finding that larger nerves require more current to achieve similar levels of activation has not been previously demonstrated. There is a strong body of literature showing that equivalent stimulation parameters can successfully activate the rat and human vagus nerves. This data is particularly compelling for the effects of VNS on memory where both rats and humans exhibit enhanced memory as an inverted-U function of current intensity with the same peak [46–49]. Our modeling efforts suggest a simple explanation for this surprising finding, which appears to lie in the use of tight-fitting stimulating electrodes for human studies and poorly fitting, oversized cuff electrodes for rat studies. When we modeled these configurations, we confirmed that identical VNS parameters can equivalently activate nerves of very different diameters under these conditions (Fig 10). This is a novel result that could substantially impact both preclinical and clinical stimulation parameters. Follow up studies comparing small diameter nerves in animals to large diameter nerves in humans should be done to confirm this finding.

While the present study focused on the evaluation of flat electrodes inside an insulating cuff for VNS, many other nerve stimulation applications utilize different stimulation methods suitable for activation of the target nerve. Some examples include wire-like devices inserted percutaneously and penetrating intrafascicular electrodes [50,51]. Electrodes implanted percutaneously provide stimulation from a single side of the nerve, and have been effectively used for multiple applications including stimulation of the occipital nerve for migraines and peripheral nerve stimulation for chronic pain [52,53]. Some of these designs use paddle electrodes that are comparable to the flat electrodes used in this study, but without an insulating cuff. Their demonstrated efficacy supports the viability of flat electrodes used in an implanted cuff electrode. However, electrodes implanted percutaneously are susceptible to migration, especially in highly mobile body areas such as the neck. Implanted cuff electrodes may be necessary to prevent migration. Intrafascicular electrodes are capable of highly selective stimulation, which may be useful for future applications of VNS by avoiding unwanted side effects and only stimulating the desired fascicles [51]. Future studies comparing each of these methods may provide new avenues to stimulate the vagus nerve.

An important consideration with neurostimulation implants is the maximum intensity that can be safely delivered. For macroelectrodes, such as the ones in this study, this value is typically defined using the Shannon equation with a k-value between 1.5 and 2.0 [54]. This equation compares the charge per phase with the charge density per phase and can be used to approximate the maximum safe stimulation level an electrode with a known surface area can deliver before causing tissue damage. Given that flat electrodes have reduced surface area compared to circumferential and helical designs, the charge density per phase will be higher and the maximum safe level of stimulation will be lower. For the flat electrodes used in this study, which have a width of 2 mm, the surface area is approximately 10 times lower than a helical electrode with the same thickness in the axial direction (assuming 270° of coverage on a 4.65 mm diameter human vagus) [55]. However, a conservative estimate using the Shannon equation predicts a maximum safe stimulation level of greater than 0.4 μC/phase, or greater than 4 mA with a 100 μs phase-width, which is in excess of that used for most clinical applications. Moreover, evidence evaluating stimulation-dependent damage to peripheral nerves suggests that other stimulation parameters, including lower pulse frequencies, can substantially expand the safe range of stimulation intensities [56].

These results provide a framework to guide the development of new electrode designs for vagus nerve stimulation. The difference in fiber recruitment between flat and circumferential contacts is not likely to meaningfully influence the efficacy of VNS techniques, and flat contacts may provide advantages in fabrication that will significantly reduce the cost of implantation as they can be designed using simpler methods such as printed circuit boards. Future studies examining the effects of electrode size and geometry may provide further insights into design features to optimize recruitment for VNS therapies.

## Supporting information

S1 Table Comsol model parameters. Both geometric and electrical parameters for the various models created in Comsol.
(DOCX)

S2 Table NEURON model parameters. Geometric parameters were interpolated to allow for a distribution of fiber diameters to be used. All parameters not listed are identical to those used in the MRG model [27].
(DOCX)

S1 Fig Impact of cuff inner diameter, contact separation, and cuff overhang on a 60° electrode. The effect of each variable appears similar to the effect observed with a standard 270° electrode. a) Increasing the inner diameter of the cuff (1 mm contact separation, 1 mm cuff overhang, 60°) drastically reduces recruitment. b) Increasing the distance between the two stimulating contacts (1 mm cuff inner diameter, 1 mm cuff overhang, 60°) increases recruitment. c) Increasing the amount of cuff overhang (1 mm cuff inner diameter, 1 mm contact separation, 60°) increases recruitment.
(TIF)

S2 Fig Flat electrode recruitment is comparable to helical electrode used for epilepsy. Due to the narrow amount of insulation covering the helical electrodes, the use of a complete cuff can improve recruitment. However, recruitment using flat electrodes is similar to recruitment with commonly used helical electrodes.
(TIF)

S3 Fig Modeling strength-duration curves at various fiber diameters. Fibers of various diameters were placed in the center of the rat sciatic fascicle with a standard circumferential cuff around it (1 mm cuff inner diameter, 1 mm contact separation, 1 mm cuff overhand, 270°). Thresholds were measured for each fiber at various pulse-widths. Data were fit with exponential functions. a) Threshold as a function of fiber diameter for various pulse-widths. b) Threshold as a function of pulse-width for various fiber diameters.
(TIF)

S4 Fig Individual rat sciatic recruitment curves. (TIF)

S5 Fig Individual rat vagus recruitment curves. (TIF)

S6 Fig Individual rabbit sciatic recruitment curves. (TIF)

S7 Fig Picture of implanted cuff electrode on rat sciatic nerve. (TIF)

S8 Fig Picture of implanted cuff electrode on rat vagus nerve. (TIF)

S9 Fig Pictures of implanted electrodes on rabbit sciatic nerve. a) Circumferential electrode around the rabbit sciatic nerve. b) Flat electrode under the rabbit sciatic nerve. Insulating cuff not shown.
(TIF)

S1 File Data from rat sciatic nerve stimulation experiments. (ZIP)

S2 File Data from rat vagus nerve stimulation experiments. (ZIP)

S3 File Data from rabbit sciatic nerve stimulation experiments. (ZIP)
