# Supplementary material for: Flat electrode contacts for vagus nerve stimulation
Source: PLoS One. 2019 Nov 18;14(11):e0215191. doi: 10.1371/journal.pone.0215191 (PMC6862926; doi:10.1371/journal.pone.0215191)
Supplement: S1 Table — Both geometric and electrical parameters for the various models created in Comsol. (DOCX) [file pone.0215191.s001.docx]

| Geometric Parameters | Rat Sciatic | Rat Vagus | Rabbit Sciatic |
| --- | --- | --- | --- |
| Nerve Diameter | 0.9 mm | 0.4 mm | 3 mm |
| Fascicle Diameter | 0.62 mm | 0.194 mm | 2.072 mm |
| Perineurium Thickness | 0.01 mm | 0.003mm | 0.031 mm |
| Epineurium Thickness | 0.13 mm | 0.1 mm | 0.43 mm |
| Cuff Inner Diameter | 1-2 mm | 1 mm | 3.2 mm |
| Cuff Outer Diameter | 2-3 mm | 2 mm | 5.2 mm |
| Cuff Overhang | 0.5-4.5 mm | 1 mm | 5 mm |
| Contact Shape | 30°-270° | 30°-270° | 270° or Flat (2.5 x 1.5 mm) |
| Contact Spacing | 0.25-5 mm | 1 mm | 10mm |
| Ambient Cylinder Diameter | 4 mm | 4 mm | 40 mm |
| Ambient Cylinder Length | 20 mm | 20 mm | 120 mm |

| Conductivity | S/m |
| --- | --- |
| Fascicle | {0.08 radial, 0.5 axial} |
| Perineurium | 0.00336 |
| Epineurium | 0.008 |
| Cuff | 2x10^-10^ |
| PI Contacts | 9x10^6^ |
| Saline | 2 |
| Fat | 0.04 |
