# Supplementary material for: Flat electrode contacts for vagus nerve stimulation
Source: PLoS One. 2019 Nov 18;14(11):e0215191. doi: 10.1371/journal.pone.0215191 (PMC6862926; doi:10.1371/journal.pone.0215191)
Supplement: S2 Table — Geometric parameters were interpolated to allow for a distribution of fiber diameters to be used. All parameters not listed are identical to those used in the MRG model [27]. (DOCX) [file pone.0215191.s002.docx]

| Geometric Parameters | µm |
| --- | --- |
| Fiber Diameter | $D$ (taken from normal distribution) |
| Node-node separation | $\Delta x=92.7652*D+108.9688$ |
| Number of nodes | $floor(ModelLength/\Delta x)$ |
| Number of myelin lamella | $6.3722*D+51.8226$ |
| Node length | 1 |
| Node diameter | $0.3449*D-0.1484$ |
| MYSA length | 3 |
| MYSA diameter | $0.3449*D-0.1484$ |
| MYSA periaxonal space width | 0.002 |
| FLUT length | $2.5811*D+19.5899$ |
| FLUT diameter | $0.0188*D^{2}+0.4787*D+0.1204$ |
| FLUT periaxonal space width | 0.004 |
| STIN length | $\frac{\Delta x-NodeLength-2*MYSALength-2*FLUTLength}{6}$ |
| STIN diameter | $0.0188*D^{2}+0.4787*D+0.1204$ |
| STIN periaxonal space width | 0.004 |
