# Supplementary figures and images for: Flat electrode contacts for vagus nerve stimulation
Source: PLoS One. 2019 Nov 18;14(11):e0215191. doi: 10.1371/journal.pone.0215191 (PMC6862926; doi:10.1371/journal.pone.0215191)

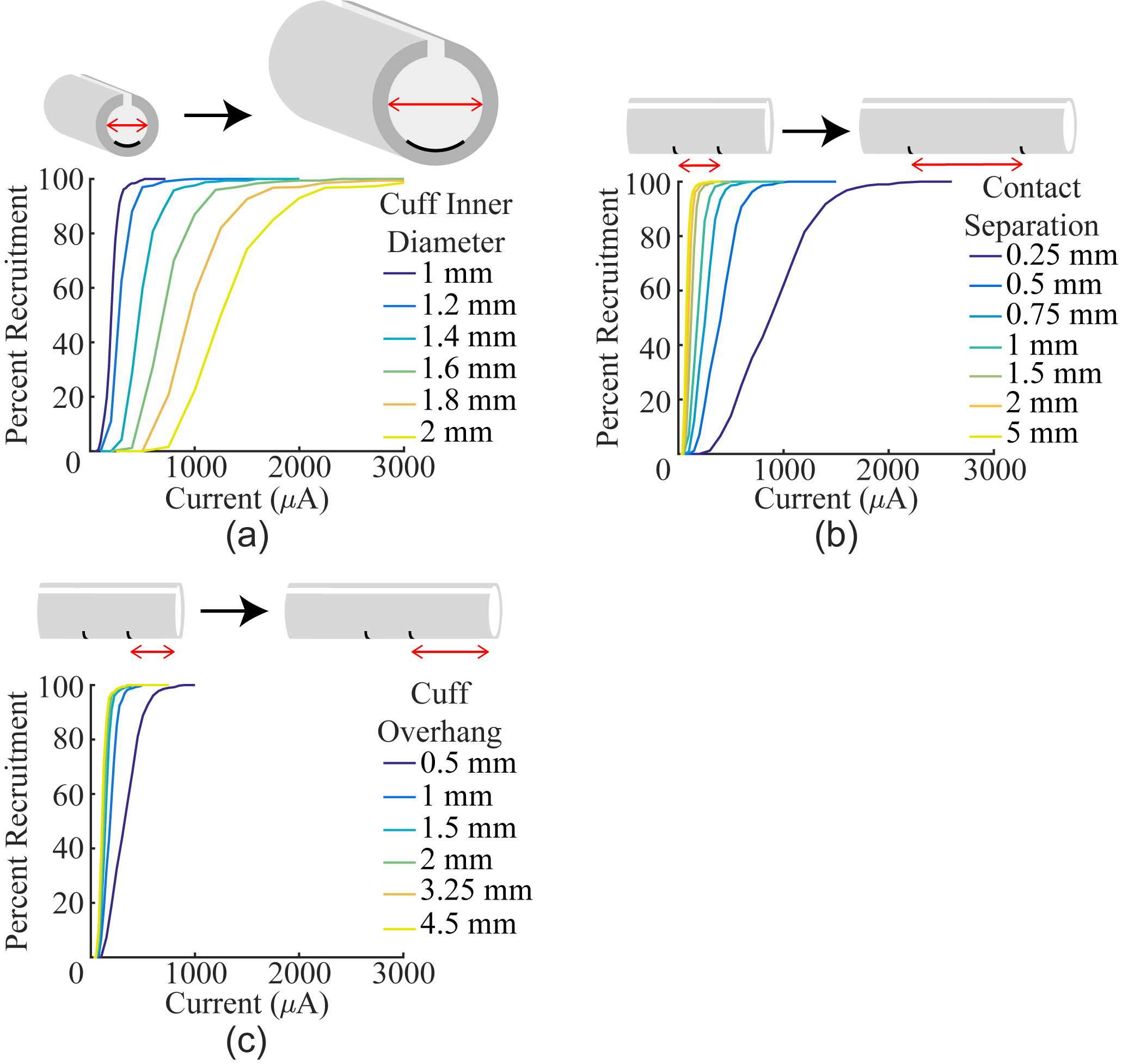

Supplement: S1 Fig — The effect of each variable appears similar to the effect observed with a standard 270° electrode. a) Increasing the inner diameter of the cuff (1 mm contact separation, 1 mm cuff overhang, 60°) drastically reduces recruitment. b) Increasing the distance between the two stimulating contacts (1 mm cuff inner diameter, 1 mm cuff overhang, 60°) increases recruitment. c) Increasing the amount of cuff overhang (1 mm cuff inner diameter, 1 mm contact separation, 60°) increases recruitment. (TIF) [file pone.0215191.s003.tif]

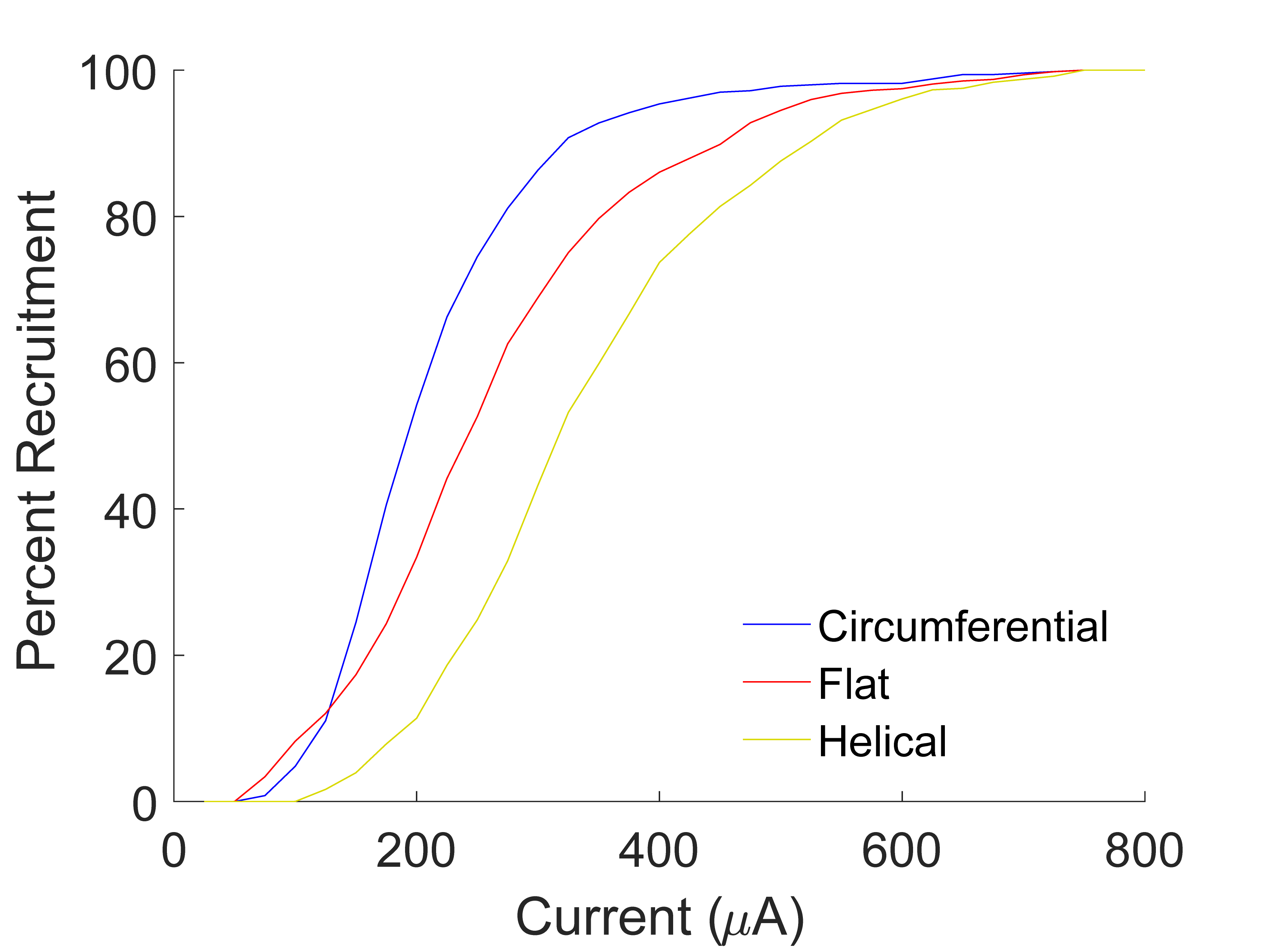

Supplement: S2 Fig — Due to the narrow amount of insulation covering the helical electrodes, the use of a complete cuff can improve recruitment. However, recruitment using flat electrodes is similar to recruitment with commonly used helical electrodes. (TIF) [file pone.0215191.s004.tif]

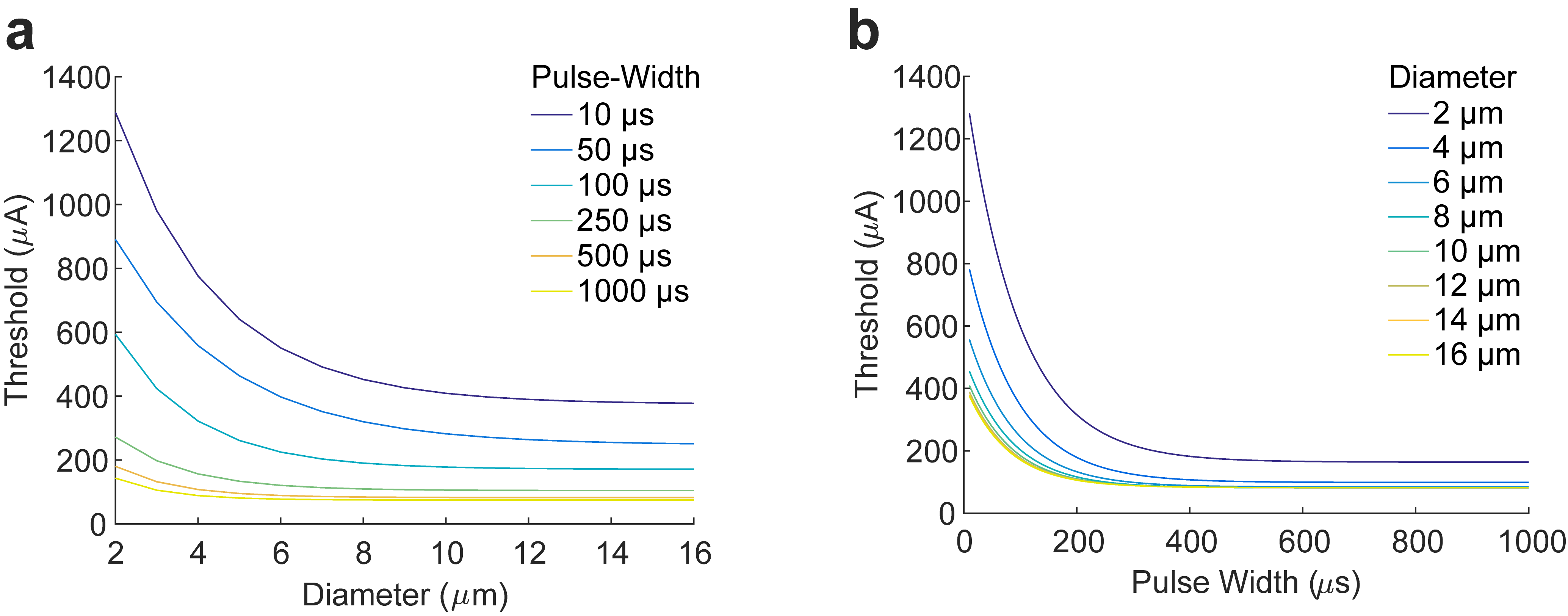

Supplement: S3 Fig — Fibers of various diameters were placed in the center of the rat sciatic fascicle with a standard circumferential cuff around it (1 mm cuff inner diameter, 1 mm contact separation, 1 mm cuff overhand, 270°). Thresholds were measured for each fiber at various pulse-widths. Data were fit with exponential functions. a) Threshold as a function of fiber diameter for various pulse-widths. b) Threshold as a function of pulse-width for various fiber diameters. (TIF) [file pone.0215191.s005.tif]

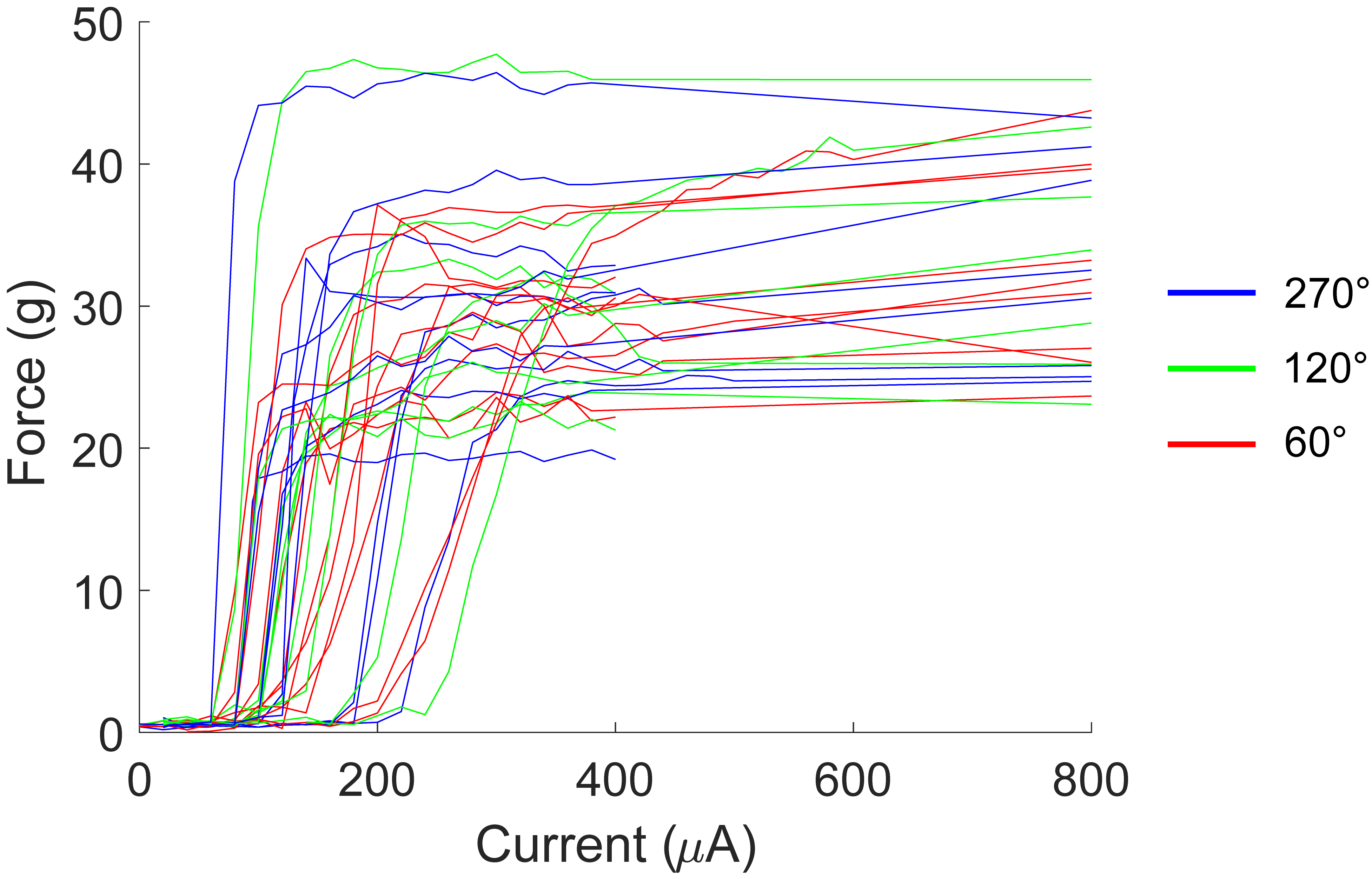

Supplement: S4 Fig — (TIF) [file pone.0215191.s006.tif]

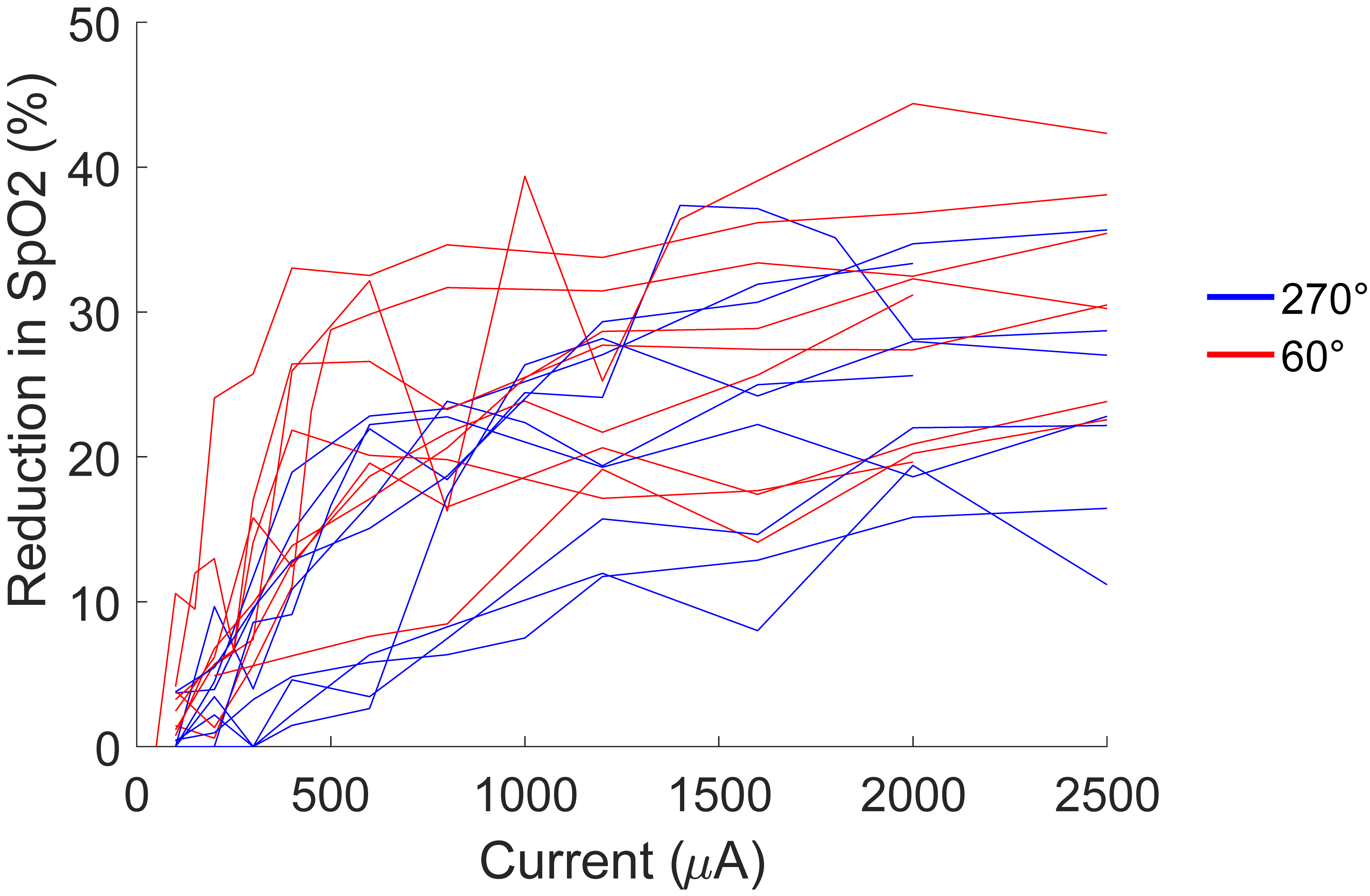

Supplement: S5 Fig — (TIF) [file pone.0215191.s007.tif]

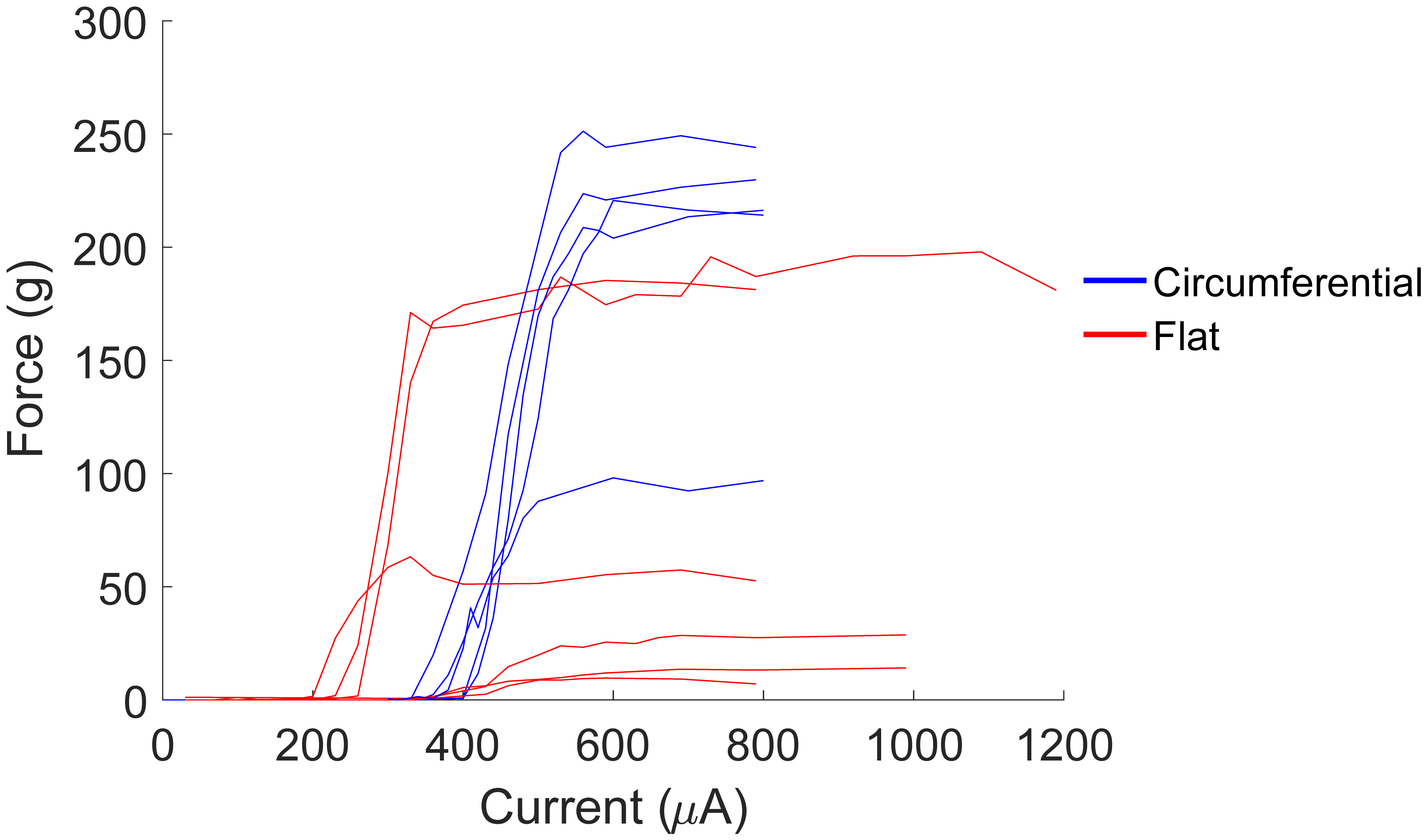

Supplement: S6 Fig — (TIF) [file pone.0215191.s008.tif]

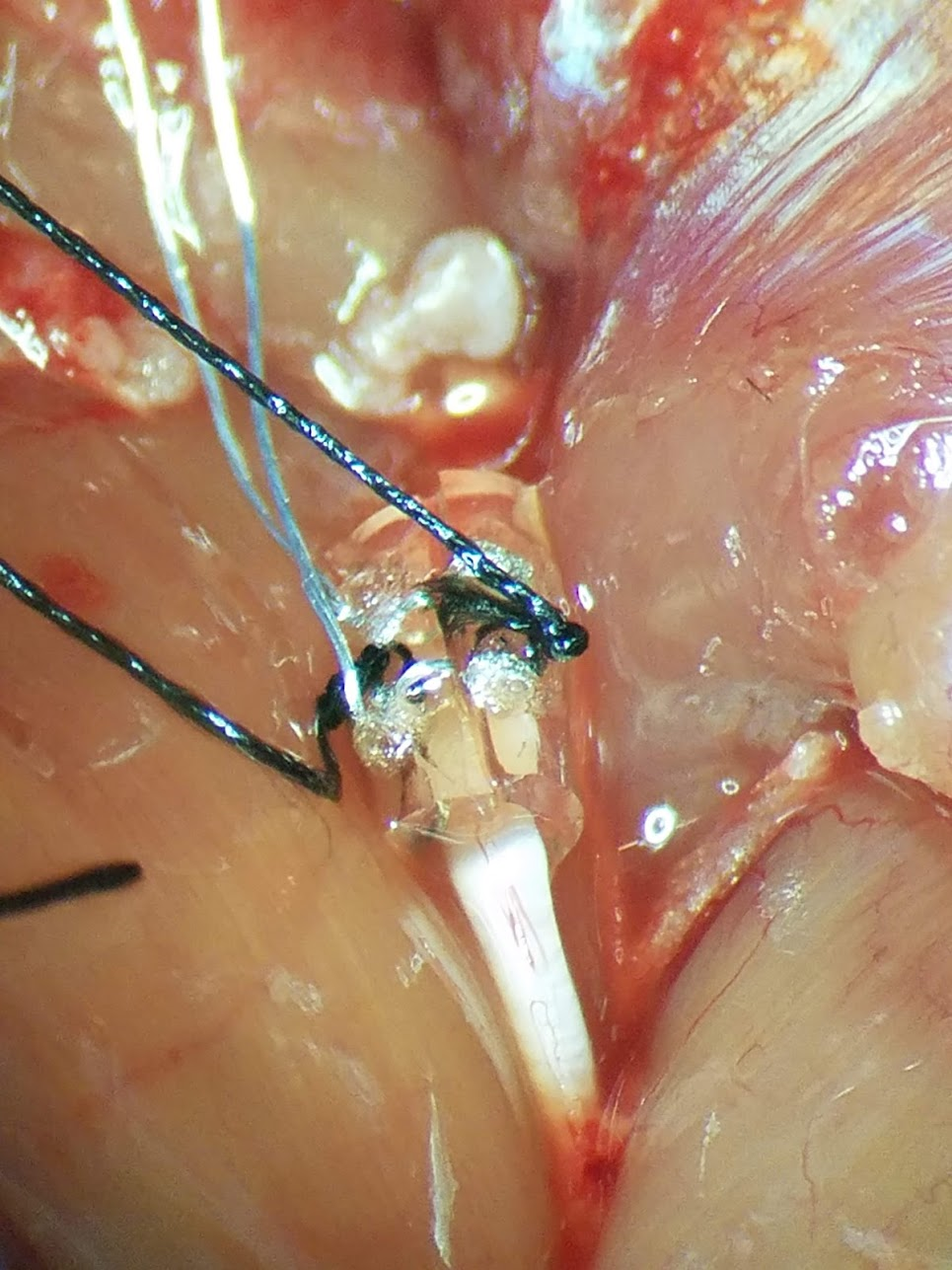

Supplement: S7 Fig — (TIF) [file pone.0215191.s009.tif]

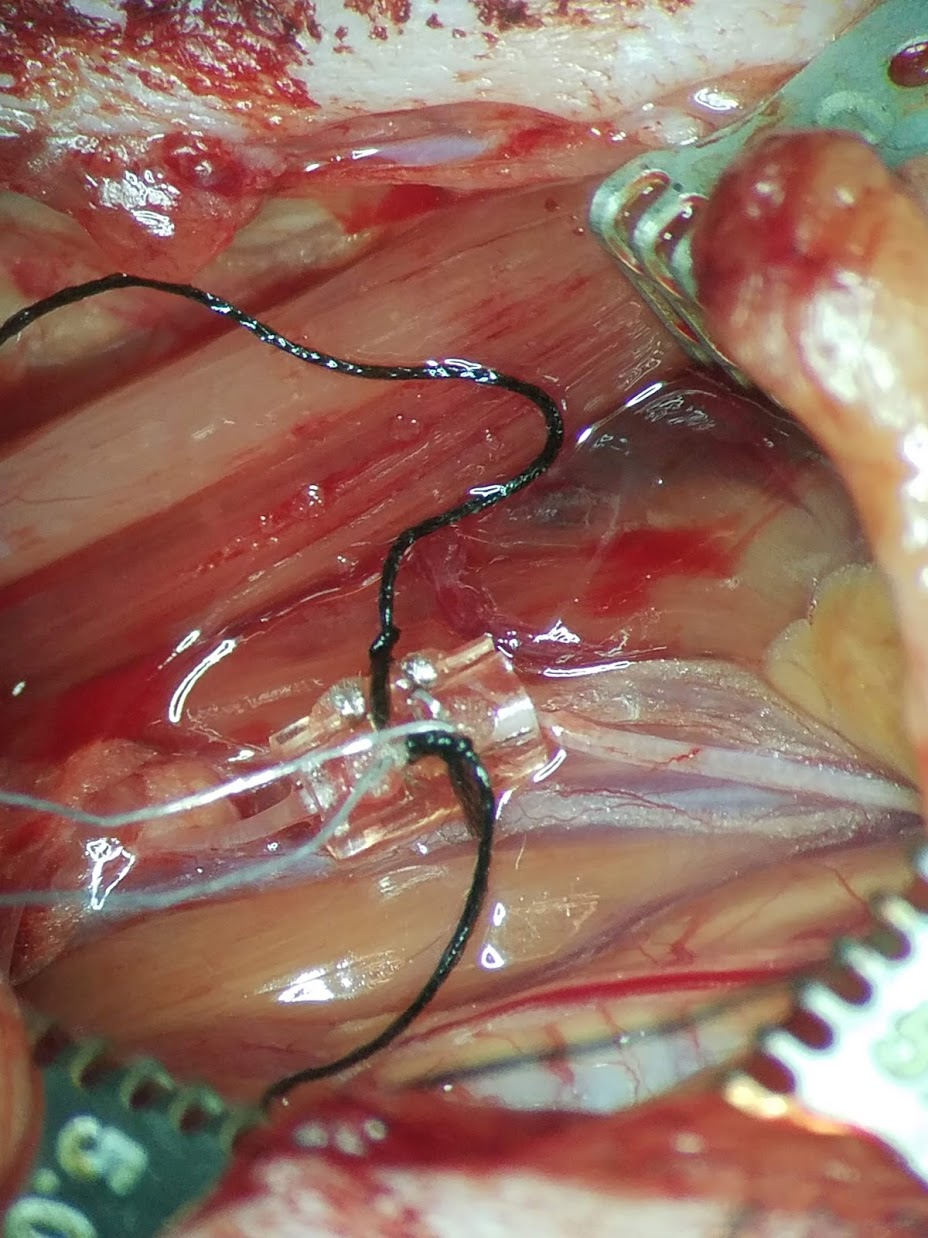

Supplement: S8 Fig — (TIF) [file pone.0215191.s010.tif]

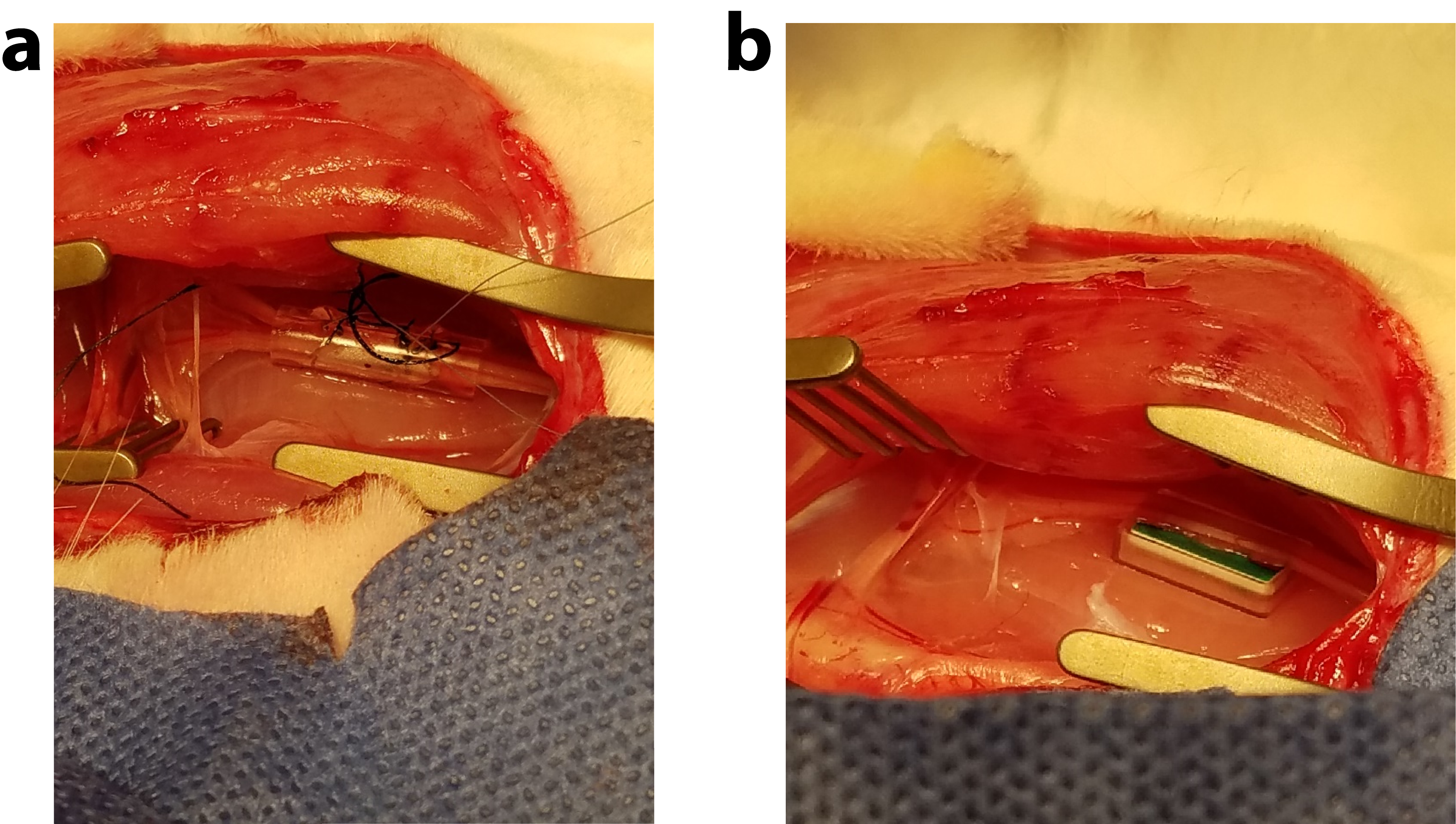

Supplement: S9 Fig — a) Circumferential electrode around the rabbit sciatic nerve. b) Flat electrode under the rabbit sciatic nerve. Insulating cuff not shown. (TIF) [file pone.0215191.s011.tif]
